# Novel Targets for Fruit Conservation Strategies Revealed by Omics Studies: A Systematic Review and Meta‐Analysis

**DOI:** 10.1155/ijfo/9963581

**Published:** 2025-10-29

**Authors:** Tatiane Timm Storch, Camila Pegoraro, Vera Quecini, Cesar V. Rombaldi, César L. Girardi

**Affiliations:** ^1^ Farroupilha Federal Institute of Education, Science, and Technology, Campus Santo Augusto, Santo Augusto, Brazil; ^2^ Plant Genomics and Breeding Center, College of Agronomy “Eliseu Maciel”, Pelotas Federal University, Pelotas, Brazil; ^3^ Embrapa Grape and Wine, Bento Gonçalves, Brazil; ^4^ Department of Agroindustrial Science and Technology, College of Agronomy “Eliseu Maciel”, Pelotas Federal University, Pelotas, Brazil

**Keywords:** berry, genomics, metabolomics, pome, postharvest, sensory, transcriptomics

## Abstract

Fresh fruit is an important dietary source of nutrients and health‐related compounds, also contributing to food security and economic development worldwide. Postharvest losses exert a huge negative impact on fruit quality, consumers′ acceptance, economic value, and market availability. High‐throughput techniques have contributed to elucidating the molecular mechanisms underlying fruit ripening and senescence. However, the application of these findings to develop conservation technologies remains scarce. The current systematic review is aimed at evaluating the literature on omics studies for sensory properties, shelf‐life duration, microbiological and physiological quality outcomes during fruit ripening, postharvest conservation, and ex planta senescence. Four databases were investigated from 2014 to 2025, and data from 171 studies were compiled, converted to Gene Ontology terms, and analyzed using multivariate methods. The results reinforced the key role of phytohormones in climacteric and nonclimacteric fruit conservation. Ethylene and abscisic acid–controlled processes are the main contributors to senescence in climacteric and nonclimacteric fruit, respectively. Among the outcomes investigated, most omics studies assessed the effects of conservation technologies on fruit quality and sensory properties. After harvest, carbohydrate and reactive oxygen metabolic pathways are important contributors to conservation strategies. Epigenetic modifications, such as DNA methylation and histones posttranslational changes, are promising targets for novel conservation techniques. Further research on the impact of conservation technologies on fruit genomic, transcriptional, and metabolic changes may contribute to devising novel, paradigm‐changing postharvest alternatives.

## 1. Introduction

Fresh fruit is an important source of nutrients and health‐related compounds, contributing to food security and economic growth worldwide. They are considered critical sources of essential vitamins, natural antioxidants, minerals, and dietary fibers, preventing and relieving nutritional ailments and deficiencies [1]. However, after harvest, fruit continues to undergo ripening and senescence processes [2], leading to physiological changes that affect their sensory, shelf life, microbiological, and metabolic properties. Several factors contribute to fruit conservation after removal from the plant, including preharvest factors, such as fertilization, irrigation, soil conditions, and plant spacing, and postharvest, transport, and storage conditions. Due to their highly perishable nature, fresh fruit and vegetables are the largest contributors to food loss and waste, reaching up to 50% of global production [3], which is estimated to represent approximately a third of world food production [3]. Thus, increasing fruit conservation would contribute to food security worldwide, promoting sustainability and reducing production costs. The most sought‐after fruit conservation outcomes may be summarized in shelf‐life period, maintenance of physiological and sensory quality, and avoidance of microbiological spoilage [3, 4]. These outcomes are mainly controlled by developmental processes associated with postripening and senescence metabolic changes, with the exception of microbiological colonization that is determined by environmental conditions after primary production and harvesting, although also influenced by endogenous developmental factors [5].

Fruits undergo several orchestrated physiological, structural, and metabolic processes from flowering to senescence, leading to the differentiation of the ovary and its associated structures into fruit, via cycles of cell division and enlargement [6, 7]. Simultaneously, fruits expand by water accumulation in the vacuoles, driven by the storage of hydrophilic compounds, and activate several metabolic pathways controlling the biosynthesis of specialized metabolites responsible for fruit sensory properties [6, 7]. Traditionally, the progression of respiration rates and hormone accumulation has been used to classify fleshy fruit ripening in climacteric and nonclimacteric. Typically, in climacteric fruit, respiration rates increase sharply, and ethylene biosynthesis is induced in the later stages of ripening (Figure 1a) [6]. In contrast, in nonclimacteric fruit, respiration rates decline toward the end of ripening, and ethylene is not required to complete the process, although responses to the phytohormone might be present (Figure 1b) [6, 7]. Abscisic acid (ABA) is hypothesized to induce ripening and senescence in nonclimacteric fruit [8, 9]. Therefore, climacteric and nonclimacteric fruits display both conserved and divergent physiological and metabolic processes during ripening and after harvest [6, 10].

Figure 1Developmental and physiological processes responsible for fruit growth, ripening, and senescence in (a) climacteric and (b) nonclimacteric fruits. Biological processes are represented by their corresponding Gene Ontology (GO) terms.

**Figure:**
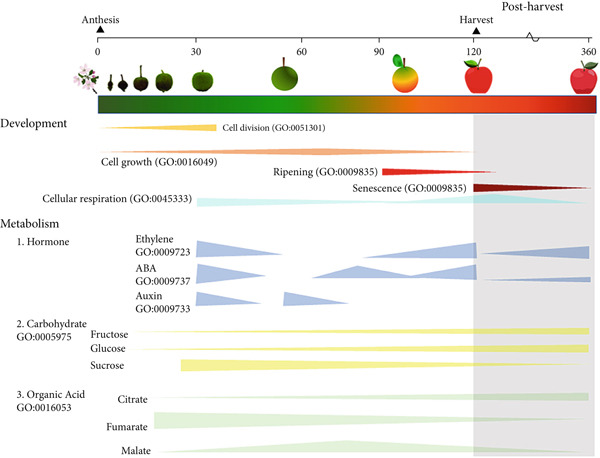
(a)

**Figure:**
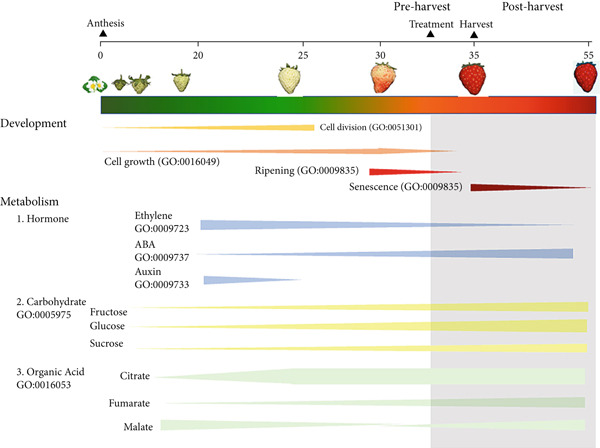
(b)

Traditionally, the processes responsible for ripening and senescence after harvest can be delayed or reduced by manipulating environmental conditions, such as management of temperature, relative humidity, and atmosphere, and by the application of chemical and/or physical treatments [3, 5, 11]. Along with temperature and atmosphere control, advanced postharvest technologies include physical treatments, such as the application of pulsed electric fields (PEFs) and cold plasma (CP), and chemical methods, such as active coating and vacuum impregnation [3, 11, 12]. The application of conservation technologies affects fruit metabolism and physiology, not only modifying targeted pathways but also interfering with biological processes that may lead to undesired consequences, such as chilling injury (CI), aroma production, rotting, and browning [12–16]. The complex nature of developmental and metabolic processes underlying fruit ripening and senescence has benefited greatly from integrated, large‐scale studies that allow concurrent surveying of thousands of information‐transmitting and effector molecules.

Recently, technical advances allowed the simultaneous investigation of the complete set of molecular players involved in biological processes via high‐throughput technologies. These technologies are collectively known as “omics” and include the global study of the genome and epigenome, transcriptome, proteome, and targeted or untargeted metabolome, along with machine learning and high‐throughput phenotyping [17, 18]. The blueprint of an organism is given by the genes in its genome and its higher order organization, determined by the chemical status of the DNA and histones, which controls its accessibility to transcription and replication machinery. The set of genes transcribed at a given time, in a specific cell or tissue, and under given conditions constitutes the transcriptome, which also includes messenger (mRNA) and small interfering RNAs (siRNAs). The products of mRNA translation are the global set of proteins or the proteome. These molecules are considered the main repositories and effectors of genetic information, which in turn control the growth, development, and metabolic processes of the organism. The metabolome consists of the whole set of metabolites in the organism, targeted to a given chemical class or global (untargeted). Other high‐throughput techniques investigate the global expression of phenotypes (phenomics), ion concentrations (ionome), lipid profiling (lipidome), and others [19]. The combination of two or more large‐scale studies is labeled “multiomics.” The integration of high‐throughput multiomics data may provide novel insights and expand our understanding of the complex physiological processes controlling fruit characteristics under postharvest conditions, contributing to enhance food security, reduce fruit loss, and promote availability and accessibility to quality products.

The knowledge on the biology of fruit ripening and senescence and the technological advances of conservation techniques increased greatly in recent years. However, biological and technological data are not unified and lack association with conservation outcomes of interest. Moreover, a significant portion of data arising from integrative studies remains detached from prospective technologies aiming at improving postharvest conservation. To contribute to narrowing this knowledge gap, the current work is aimed at systematically reviewing omics studies on fruit ripening and postharvest to determine the impact of developmental and metabolic pathways on sensory, microbiological, metabolic (quality), and shelf‐life aspects of fruit after harvest.

## 2. Methods

### 2.1. Search Strategy and Inclusion/Exclusion Criteria

Systematic review and meta‐analyses were performed according to Preferred Reporting Items for Systematic Reviews and Meta‐Analysis (PRISMA) and International Food Information Services (IFIS) Good Review Practice guidelines. Initially, we built a cache of 20 relevant articles to extract the primary search terms related to the research question. The list of terms included synonyms, alternative spellings, and truncations. Three search strings were assembled, consisting of terms related to (i) omics and high‐throughput techniques, (ii) fruit and their botanical specifications, and (iii) ripening and postharvest conservation. Strings were connected using operators “AND” and “AND/OR.” Data were collected from 2014 to 2025, from queries of Scopus, Web of Science, ScienceDirect, and PubMed databases. References were stored and managed using Zotero 6.0.26. Inclusion criteria were publication period, in peer‐reviewed scientific journals, and written in English. Studies with tomato (*Solanum* spp.) and its wild relatives, *in planta* fruit ripening and senescence, and those that did not provide primary data were excluded. A list of the 10 most representative fruit species, the investigated postharvest technologies, and the outcomes is presented in Table 1.

**Table 1:** List of fruit species, principal postharvest technologies, and conservation outcomes selected for the systematic review.

	**Fruit (scientific name)**	**Technology**	**Outcome**
Climacteric	Apple (*Malus* spp.)	Physical treatment	Shelf life (time)
	Banana (*Musa* spp.)	Atmosphere manipulation	Sensory
	Peach (*Prunus persica* L.)	Temperature manipulation	Quality (physiological)
	Pear (*Pyrus* spp.)	Chemical treatment	Microbiological
	Persimmon (*Diospyros* spp.)		

Nonclimacteric	Cherry (*Prunus avium* L.)		
	Citrus (*Citrus* spp.)		
	Cucurbitaceae		
	Grape (*Vitis* spp.)		
	Strawberry (*Fragaria* spp.)		

### 2.2. Data Selection and Processing

After an initial automated filtering for redundant and nonaccessible works, the retrieved documents were manually checked for duplicates and irrelevant results that were removed. The second round of screening retained and classified the publications according to the inclusion criteria. Manuscripts that did not provide access to primary data were eliminated. The pipeline and search results are schematically presented in Figure 2. Quality assessment was performed by appraising relevance, reliability, validity, and applicability of the evidence, and risk of bias was graphically represented using the package robvis [20] in the statistical computing environment R 4.4.1 [21]. Manuscripts with high overall risk of bias were removed from further analysis.

Figure 2(a) Schematic representation of PRISMA flow diagram for the systematic review of omics studies of fruit postharvest conservation reporting on sensory, quality, shelf life, and microbiological aspects from 2014 to the present. Summary of the investigated studies by (b) publication year and (c) country of origin. (d) Pie chart of the species of climacteric and nonclimacteric fruit and type of omics study for (e) climacteric and (f) nonclimacteric fruits.

**Figure:**
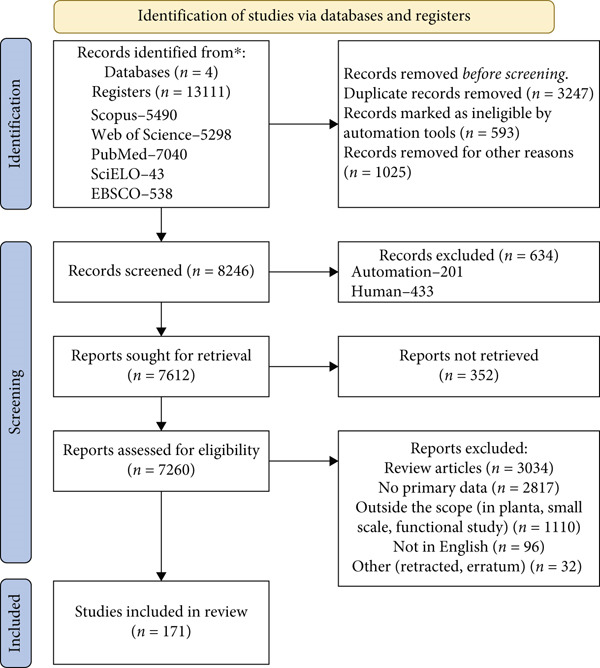
(a)

**Figure:**
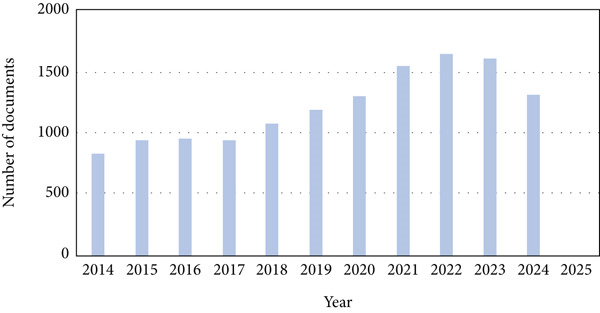
(b)

**Figure:**
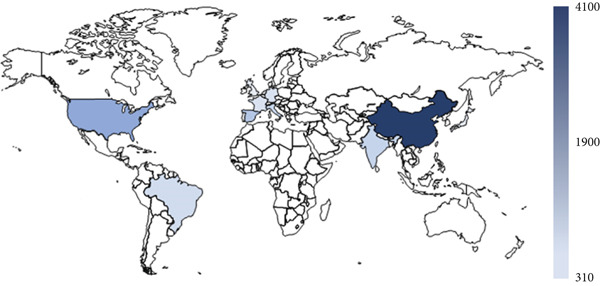
(c)

**Figure:**
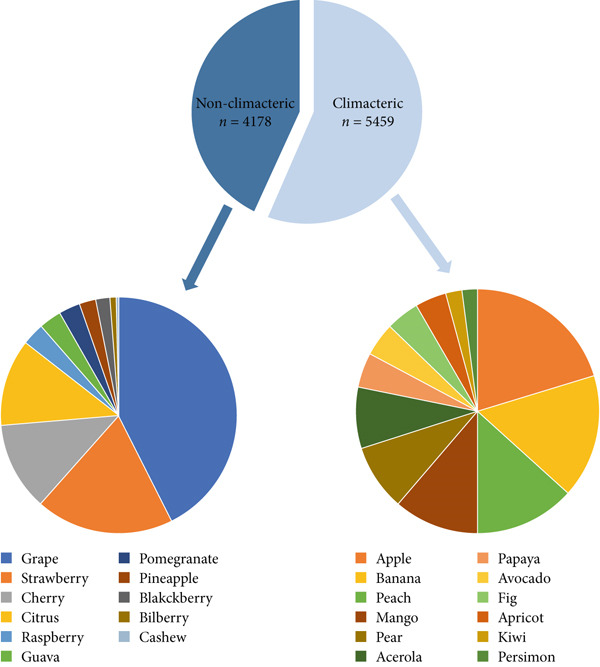
(d)

**Figure:**
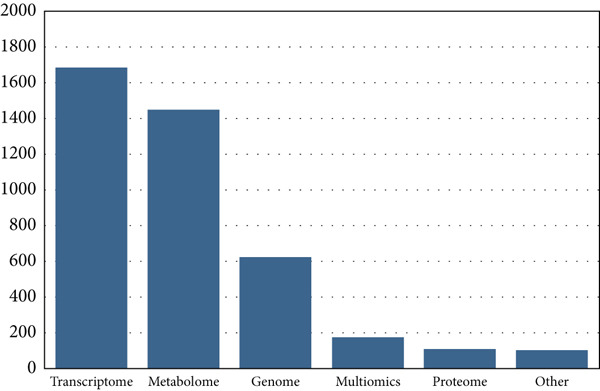
(e)

**Figure:**
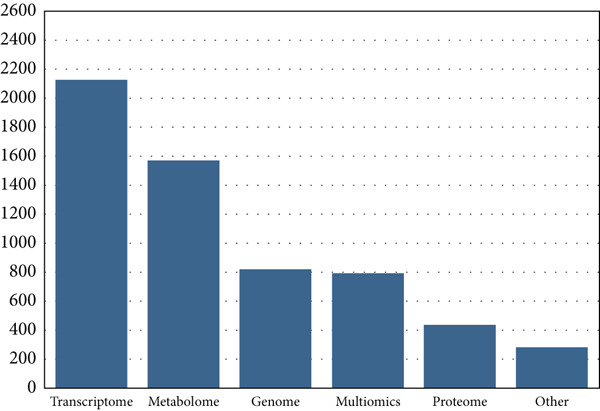
(f)

### 2.3. Evidence Synthesis and Meta‐Analyses

Data synthesis was performed using a structured framework consisting of four outcomes: (1) sensory, (2) shelf‐life, (3) microbiological, and (4) physiological quality for climacteric and nonclimacteric fruits (Tables 2 and 3). Evidence tables were built including the outcome, omics method, experimental design, and a representation of the direction of the impact (positive or negative) on each outcome. The categories and classes in the evidence table were constructed using Gene Ontology (GO) terms [160]. GO terms for genes characterized by Kyoto Encyclopedia of Genes and Genomes (KEGG) pathways were retrieved using KEGGSOAP [161] in R 4.4.1. Categories included in the evidence table were defined according to the GO category under “Biological Process” and “Cellular Component” classifications. Significance levels were retrieved from the original publications. The studies were weighed according to their reliability to provide valid estimates of cumulative information. Inverse variance was used to measure the study precision, consisting of a composite calculation of the global variance and sample size; therefore, studies with larger sample sizes and smaller experimental errors were considered more reliable and received higher weights. To maintain the inferential properties of the studies, global data was normalized by logarithm transformation.

**Table 2:** Summary of studies with climacteric fruit included in the analyses. The study target, used omics techniques, and outcome are described.

**Study**			**Outcome**	**Reference**
**Fruit**	**Target**	**Omics technique**		
Apple				
	Firmness loss during storage is associated with expression of an *α*‐L‐arabinofuranosidase regulated by ethylene	Transcriptome, phenomics (physicochemical and sensory analyses)	Quality, sensory, shelf life	[22]
	Changes in peel plastid structure during ripening	Phenomics (ultrastructural analyses)	Sensory, quality, shelf life	[23]
	Conservation techniques exert distinct effects on gene expression	Transcriptome, phenomics (physicochemical and sensory analyses)	Quality, shelf life	[24]
	Xyloglucan endotransglucosylase/hydrolase gene family expression during ripening and softening	Transcriptome	Sensory, quality, shelf life	[25]
	Apple texture in multifamilies using genome‐wide association (GWA)	Genome (GWA)	Quality, shelf life	[26]
	Hormone‐controlled anthocyanin production after harvest	Transcriptome	Sensory, quality	[27]
	Overexpression of an anthocyanin regulator basic helix–loop–helix (bHLH) transcription factor accelerates ethylene biosynthesis and senescence	Genome (functional analyses)	Sensory, quality, shelf life	[28]
	Association between anthocyanin accumulation and internal browning flesh disorder	Functional analyses (gene overexpression, metabolome, gene expression)	Sensory, quality, shelf life	[29]
	Expression analyses in individuals with distinct crispness	Genome, transcriptome	Sensory, quality	[30]
	Effect of ozone and 1‐MCP on superficial scald disorder during cold storage	Transcriptome, proteome, metabolome	Sensory, quality, shelf life	[31]
	1‐MCP prevents the expression of ripening‐related genes	Transcriptome	Quality, shelf life	[32]
	Effect of hormones during early ripening	Transcriptome	Sensory, quality	[33]
	Effect of NAC (NAM, ATAF1/2, CUC2 family) transcription factors on flavor ester biosynthesis	Genome (methylation), transcriptome	Sensory, quality	[34]
	Evolution of volatiles and gene expression during ripening	Transcriptome, metabolome	Sensory, quality	[35]
	Senescence is delayed by not prevented by conservation techniques	Transcriptome, phenomics (physicochemical and sensory analyses)	Sensory, quality, shelf life	[36]
	Effect of ethylene responsive factors on histone deacetylase expression during ripening	Genome (chromatin immunoprecipitation sequencing—ChIP‐Seq), transcriptome (chromatin immunoprecipitation—quantitative reverse transcription polymerase chain reaction, ChIP‐qRT‐PCR), functional analyses (heterologous expression)	Quality, shelf life	[37]
	Effect of wax coating on gene expression	Transcriptome	Quality, shelf life	[38]
	Calcium represses ethylene biosynthesis during apple fruit ripening by regulating posttranslational status of an APETALA2/ETHYLENE RESPONSE FACTOR (AP2/ERF) protein	Gene expression, (RT‐qPCR and ChIP‐RT‐qPCR) functional analyses	Quality, sensory, shelf life	[39]
	Calcium represses ethylene biosynthesis during apple fruit ripening by regulating posttranslational status of an AP2/ERF protein	Gene expression, (RT‐qPCR and ChIP‐RT‐qPCR) functional analyses	Sensory, quality, shelf life	[40]
	Effect of ethylene on malate transport via transcription factors network	Transcriptome, functional analyses (yeast‐2‐hybrid—Y2H, luciferase promoter fusion—Luc pro, ectopic expression)	Quality, shelf life	[41]
	Regulation of volatile ester biosynthesis during ripening	Transcriptome, metabolome	Sensory, quality	[42]
	Identification of a zinc finger homeodomain transcription factor that induces expression of a *β*‐galactosidase	Transcriptome, functional analyses (Y2H, Luc pro, ectopic expression)	Sensory, quality, microbiological	[41]
	Overexpression of a polygalacturonase gene affects fruit development and structure	Functional analyses (overexpression), transcriptome	Quality, shelf life, microbiological	[43]
	Mechanism regulating the formation of watercore during ripening	Transcriptome, functional analyses (Y2H, Luc pro, ectopic expression)	Quality, shelf life	[44]
	Effect of DNA methylation on gene expression in two distinct ripening stages	Genome (bisulfide sequencing), transcriptome, metabolome (sugars and hormones)	Quality	[45–47]
	Mobile mRNA from seed to flesh induces ripening	Genome (resequencing), transcriptome, functional analyses	Shelf life	[45–47]
	NAC and WRYK (WRKYK domain) transcription factors control fruit softening via ethylene mediated regulation of an xyloglucan endotransglucosylase/hydrolase	Transcriptome, functional analyses (ectopic expression, Y2H, Luc)	Quality, shelf life	[45–47]
	Effects of aminoethoxyvinylglycine and 1‐MCP on gene expression and quality traits	Transcriptome	Quality, shelf life	[48]

Banana				
	Expression of *AUXIN RESPONSE FACTOR* (*ARF*) genes and metabolite profiling after harvest	Transcriptome, metabolome	Shelf life	[49]
	General metabolite database, ripening as case study	Genome, transcriptome	Sensory, quality	[50]
	General genomics database, ripening as case study	Genome, transcriptome	Sensory, quality	[51]
	Expression of *POLYPHENOL OXIDASE* (*PPO*) gene family	Transcriptome	Sensory, quality, microbiological	[52]

Peach				
	Effect of gibberellin pretreatment on cold storage	Transcriptome	Sensory, quality, shelf life	[53]
	Acid accumulation	Multiomics (GWA mapping and transcriptome)	Quality	[54]
	Biological activity (flavonoid)	Metabolome, genomics	Quality	[55]
	Aroma	Multiomics (metabolome, genomics, transcriptome)	Quality	[56]
	Chilling injury	Metabolome, transcriptome, genome	Shelf life, quality	[57]

Pear				
	Russeting	Proteome, transcriptome	Quality	[58]
	Russeting	Metabolome, transcriptome, proteome	Quality	[59]
	Ripening	Metabolome, proteome, transcriptome, DNA methylome, small RNAome	Quality, shelf life	[60]

Persimmon				
	Seedlessness	Phenomics, convolutional neural networks	Quality	[61]
	Chilling injury	Transcriptome, convolutional neural networks	Shelf life, quality	[62]

**Table 3:** Summary of studies with nonclimacteric fruit included in the analyses. The study target, used omics techniques, and outcome are described.

**Study**			**Outcome**	**Reference**
**Fruit**	**Target**	**Omics technique**		
Cherry				
	Cold storage and 1‐MCP	Metabolome	Shelf life	[63]
	*Penicillium expansum* responses	Genomics	Quality, shelf life	[64]
	Maturity and quality quantitative trait loci (QTL) mapping	Genomics (GWA mapping)	Quality, shelf life	[65]
	Germplasm carbohydrate profiling	Metabolome	Quality	[66]
	Effect of ozone treatment on cherry proteome after harvest	Proteome	Shelf life	[67]
	Fruit thinning, orchard management	Phenomics	Quality, shelf life	[68]
	Lipoxygenase‐encoding gene families during ripening	Genome, transcriptome, metabolome	Quality	[69]
	Effect of chitosan coating on metabolism in cold storage	Phenomics	Shelf life	[70]

Citrus				
	Ripening and senescence	Transcriptome, metabolome	Shelf life	[71]
	Peel texture	Transcriptome, metabolome	Quality	[72]
	Nonchilling peel pitting	Transcriptome	Shelf life	[2]
	Mold suppression by salicylic acid (SA) and cinnamon	Transcriptome	Quality, shelf life	[73]
	Cinnamaldehyde on phenylpropanoid pathway	Transcriptome, metabolome	Shelf life	[74]
	Water transport, wax biosynthesis	Transcriptome, metabolome	Shelf life	[75]
	SA effect on cell wall metabolism	Transcriptome, metabolome	Quality, shelf life	[76]
	Wax coating cold storage	Transcriptome, metabolome	Quality, shelf life	[77]
	Anthocyanin accumulation during drought	Metabolome	Quality, Shelf life	[78]
	Puffing disorder	Phenomics, metabolite profiling	Quality, shelf life	[79]
	Flavonoid biosynthesis	Genome, transcriptome, metabolome, virus‐induced gene silencing (VIGS)	Quality	[80]
	Bioactive compounds	Genomics, transcriptome, metabolomics	Quality	[81]

Cucurbitaceae				
	Expression of abscisic acid (ABA) pathway genes during ripening	Transcriptome	Quality	[82]
	Long noncoding RNA in ripening	Transcriptome	Shelf life	[83]
	Response to biocontrol agent	Transcriptome, proteome	Shelf life	[84]
	Silencing of a gene encoding an ascorbate oxidase	Genetic engineering	Quality, shelf life	[85]
	Sugar metabolism in contrasting genotypes	Transcriptome	Shelf life	[86]
	Flavonoid biosynthesis, nematoid resistance	Transcriptome, proteome	Quality, shelf life	[87]
	Aroma volatiles during ripening	Transcriptome, metabolome	Quality	[88]
	Transcriptome of near‐isogenic lines (NILs) with high flesh firmness	Transcriptome	Shelf life	[89]
	Pericarp color, flavonoid accumulation	Genome (QTL mapping), metabolome	Quality	[90]
	Aroma volatiles, flesh color in watermelon germplasm	Genome (GWA), metabolome	Quality	[91]
	Bitterness	Genome, metabolome	Quality	[92]
	Heat shock proteins expression under abiotic stresses	Transcriptome, metabolome	Quality	[93]
	Database, breeding	Genome, transcriptome	Quality, shelf life	[94]
	Ozone effect on phenylpropanoid biosynthesis	Metabolome, enzyme activity, gene expression	Quality, shelf life	[95]
	Ripening regulation in contrasting lines	Genome (QTL mapping), genome editing (clustered regularly interspaced short palindromic repeats/CRISPR‐associated Protein 9, CRISPR/Cas9), transcriptome, DNA affinity and DNase sequencing	Shelf life	[96]
	Fruit size, fruit color	Transcriptome, metabolome	Quality	[97]
	Flesh firmness	Genome (QTL mapping), bulk segregant analysis sequencing, gene function (yeast 1‐hybrid—Y1H, dual luciferase)	Shelf life	[98]

Grape				
	Ultraviolet influence on stilbene biosynthesis	Metabolome, transcriptome	Quality	[99]
	Berry ripening under water stress	Metabolome, transcriptome	Quality	[100]
	Specialized metabolism expression during ripening	Transcriptome	Quality	[101]
	Gene expression networks during ripening	Transcriptome, metabolome	Quality	[102]
	Anthocyanin biosynthesis	Transcriptome, metabolome	Quality	[103]
	MicroRNA (miRNA) regulation during ripening	Transcriptome	Quality	[104]
	Flavonoid variation in grape germplasm	Transcriptome, metabolome	Quality	[105]
	Copper stress during fruit ripening	Transcriptome, proteome, metabolome, and miRNAome	Quality, shelf life	[106]
	Red blotch virus infection during ripening	Transcriptome, metabolome	Quality, shelf life	[107]
	Abiotic and biotic stresses during ripening	Transcriptome, metabolome	Quality, shelf life	[108]
	Grafting on phenolic compounds accumulation in berry skin	Transcriptome, metabolome	Quality	[109]
	Virus infection during ripening	Transcriptome, metabolome	Quality	[110]
	Effect of a deacetylase inhibitor during ripening	Transcriptome, proteome	Quality	[111]
	Microbiota effect on chemical profile	Genome, metabolome	Quality, shelf life	[112]
	Anthocyanin accumulation during ripening	Transcriptome, metabolome	Quality	[113]
	Berry decay, shriveling, and weight loss	Genome (QTL and GWAS mapping), phenomics	Shelf life	[114]
	Sugar transporter gene family expression	Transcriptome	Quality	[115]
	Cadmium stress with distinct rootstocks	Transcriptome, metabolome	Quality	[116]
	Fungicide effect on berry gene expression and chemical profile	Transcriptome, metabolome	Quality, shelf life	[117]
	Effect of microcapsules for berry conservation	Phenomics	Quality, shelf life	[15]
	Treatment with jasmonates on berry gene expression and metabolic profile	Transcriptome, metabolome	Quality	[118]
	Chitosan coating during postharvest	Phenomics	Shelf life	[119]
	Nanomicroplastics in grape seedlings	Transcriptome, metabolome	Quality, shelf life	[120]

Strawberry				
	Light and ABA regulation of anthocyanin production	Transcriptome	Quality	[121]
	Flavonoid and phenylpropanoid accumulation during ripening	Transcriptome	Quality, shelf life	[122]
	Regulation of eugenol production in ripe receptacles	Transcriptome, metabolome	Quality	[123]
	Flavonoid pathway genes	Transcriptome, network analyses	Quality	[124]
	Flavonoid and anthocyanin biosynthesis during ripening	Proteome	Quality	[125]
	Effect of exogenous auxin and abscisic acid	Transcriptome	Quality, shelf life	[126]
	Silencing of a *β*‐galactosidase gene	Transcriptome, genetic engineering	Quality, shelf life	[127]
	Changes in cell wall components during ripening	Metabolome	Quality, shelf life	[127]
	Effect of light and temperature on aroma formation	Transcriptome, metabolome	Quality	[128]
	Effect of chitosan coating during harvesting	Transcriptome	Quality, shelf life	[129]
	Role of oxidative phosphorylation during ripening	Transcriptome	Quality, shelf life	[130]
	Gene expression and metabolite accumulation during ripening	Transcriptome, metabolome	Quality	[131]
	Regulation of RNA‐directed DNA methylation during ripening	Methylated DNA sequencing	Quality, shelf life	[132]
	Berry metabolome during ripening	Metabolome	Quality, shelf life	[133]
	Effect of alginate oligosaccharide after harvest	Transcriptome, metabolome	Quality, shelf life	[134]
	Ectopic expression of a tonoplast‐localized vacuolar phosphate transporter improves postharvest traits	Transcriptome, genetic engineering (overexpression)	Quality	[135]
	Ectopic expression of xyloglucan endotransglucosylase/hydrolase encoding genes accelerate ripening	Transcriptome, genetic engineering (overexpression)	Quality	[136]
	Effect of monochromatic light during fruit ripening	Transcriptome, metabolome	Quality, shelf life	[137]
	Effect of biocontrol agent on fruit quality after harvest	Metabolome, proteome	Quality, shelf life	[138]
	Gene expression in fruit with distinct storability	Transcriptome	Shelf life	[139]
	Characterization and gene expression of *PECTIN METHYLESTERASE* genes during ripening	Genome, transcriptome	Quality, shelf life	[140]
	Expression of endoxylanase encoding genes in cultivars with different flesh softening	Transcriptome	Quality, shelf life	[141]
	Effect of chitosan on *Botrytis cinerea*–infected fruit	Metabolome, proteome	Quality, shelf life	[142]
	Profiling of mRNA methylation during ripening	Transcriptome (RNA and N6‐methyladenosine, m6A‐sequencing), functional analyses (Y2H, Luc pro, in vivo transient expression)	Quality, shelf life	[143]
	Effect of DNA and histone methylation on ripening	Transcriptome, proteome	Quality, shelf life	[144]
	Effect of cold on anthocyanin accumulation	Transcriptome, functional analyses (Y2H, Luc pro in vivo transient expression)	Quality, shelf life	[145]
	Gene expression and metabolite profiling during *in planta* and *off planta* ripening	Transcriptome, metabolome	Quality, shelf life	[146]
	Gene expression and metabolite profiling in a mapping population	Genome (QTL and GWAS mapping), transcriptome, metabolome	Quality	[147]
	WRYK transcription factor activates expression of a *PECTATE LYASE* gene	Transcriptome, genetic engineering	Quality, shelf life	[148]
	Jasmonate (JA) treatment and *Botrytis cinerea* infection	Transcriptome	Quality, shelf life	[149]
	Effect of preharvest treatments on conservation	Metabolome, transcriptome	Quality, shelf life	[150]
	Methylation inhibition after harvest	Transcriptome	Quality, shelf life	[151]
	Octoploid wild species	Genomics (de novo assembly)	Quality	[152]
	Exogenous melatonin delays ripening by affecting ABA signaling	Transcriptome, phenomics (biochemistry, morphology)	Quality, shelf life	[153]
	Gene expression in natural and transgenic cell wall mutants	Transcriptome, genetic engineering (gene silencing)	Quality, shelf life	[154]
	Coordination of aroma formation and anthocyanin production	Transcriptome, metabolome	Quality, shelf life	[155]
	Effect of nanoselenium application in fungicide treated fruit	Transcriptome, metabolome	Quality, shelf life	[156]
	Patterns of histone modifications control ripening	Genome (ChIP‐Seq), transcriptome, small RNA (sRNA) sequencing	Quality, shelf life	[157]
	Silencing of a *RHAMNOGALACTURONAN LYASE* gene retains firmness after harvest	Genome, transcriptome, metabolome, genetic engineering (gene silencing)	Quality, shelf life	[158]
	Loss of function mutation of an anthocyanin reductase activates anthocyanin biosynthesis	Genome (bulk segregant analysis sequencing, BSA‐seq), transcriptome, metabolome	Quality, shelf life	[159]

GO data from the manuscripts were used in global multivariate analyses, using term frequency, dispensability, and uniqueness. The association between the co‐occurring GO terms and the outcomes was investigated by clustering analyses using z‐score normalized data retrieved from the publications. The adjacency matrix between the GO terms and the outcomes was used to construct the relevance network for all the data, using a threshold of 0.50, with igraph [162] in R. Logistic regression model (binomial) between GO terms and postharvest conservation strategies and odds ratio (OR) were calculated in R. The investigated outcomes have different time span and quantitative measures for the investigated fruit species; therefore, to provide a broader perspective on the influence of developmental and physiological process, regression modes were generated for each fruit. Multivariate analyses were carried out using sparse partial least square–discriminant analysis (sPLS‐DA) for the biological process GO terms using the fruit species as discriminant with the mixOmics package [163] in R 4.4.1. The conservation methods used in the studies included in the meta‐analysis were evaluated according to the technology readiness level (TRL) of strategy, estimated based on a scale from 1 to 9 with 1 being the least and 9 most mature technology, using an online tool (TRL Calculator) developed by the European Space Agency (ESA) and available online (http://trlcalculator.esa.int/).

## 3. Results

### 3.1. Systematic Review

The literature searches identified a total of 13,111 articles from four investigated databases. Before conducting a detailed screening, duplicate and ineligible records were removed, resulting in 8246 (62.9%) records submitted for automated and manual investigation. From these, a total of 634 (7.7%) records were removed, and a further 352 (4.3%) records could not be retrieved, resulting in 7260 (88%) records assessed for eligibility (Figure 2a). Individual analyses of these records resulted in the elimination of review articles, publications without primary data or outside the scope, written in languages other than English, or other motives, such as retraction. The remaining publications were investigated by relevance, reliability, validity, applicability of the evidence, and risk of bias (Figure S1), and 171 studies matching the inclusion criteria were considered in the meta‐analyses.

In the period investigated, the number of published omics studies assessing fruit postharvest increased linearly, reaching 1633 papers in 2022 (Figure 2b). Globally, China was the country with the highest number of publications in the period, producing more than 4000 articles on the subject in 10 years, followed by the United States, Spain, and Italy (Figure 2c). India and Brazil were the countries from the global south producing the highest number of articles in the period (Figure 2c). The number of articles investigating climacteric fruit was slightly higher (5459, 56.6%) than those on nonclimacteric (4178, 43.4%), not considering the studies investigating both fruit types (Figure 2d). Considering that work with tomato (*Solanum* spp.) and its wild relatives was not included, apple was the most frequently investigated climacteric fruit, followed by banana and peach, whereas grapes and strawberries were the most prevalent nonclimacteric fruit in omics studies (Figure 2d). For both classes of flesh fruit, transcriptome studies were the most frequent, followed by metabolome in climacteric (Figure 2e) and genome in nonclimacteric fruit (Figure 2f). Integrative, multiomics studies were more frequently retrieved for nonclimacteric fruit (Figure 2f). Tables 2 and 3 summarize the studies included in the meta‐analyses, describing the fruit species, biological questions, the used omics techniques, and the conservation outcome for climacteric and nonclimacteric fruit, respectively. Further details on the studies included in the meta‐analysis and their association with the outcomes investigated are presented in the following sections.

### 3.2. Temperature Control

Temperature is considered one of the most important external factors affecting fruit ripening and postharvest conservation [164, 165]. Accordingly, most studies included in the systematic review discuss the effect of temperature on postharvest conservation (Tables 2 and 3). Climacteric fruit undergo rapid physiological decay and microbiological contamination during transportation and storage at ambient temperature [166]. Apple, pear, and stone fruit are the prevalent species in postharvest studies investigating the effects of temperature on conservation (Table 2). High‐throughput studies demonstrated that postharvest conservation under low temperature affects ethylene, auxin, and gibberellin signal transduction pathways, cell wall enzyme metabolism, specialized metabolism pathways, and abiotic stress response pathways in climacteric fruit ([164, 165]; [24]).

Although not as susceptible to high temperature‐induced quality loss, nonclimacteric fruit displays several physiological modifications under low temperature [167]. A smaller number of studies investigated the effects of temperature on nonclimacteric fruit (Table 3). In this class of fruit, cold storage after harvest mostly affects biotic and abiotic stress pathways and primary metabolic processes, such as gluconeogenesis and starch biosynthesis, photosynthesis, translation and processing of mRNA, intracellular lipid transport, protein posttranslational processing, and intracellular membrane trafficking [2, 69, 93].

Extensive transcriptional reprogramming caused by temperature brings about developmental and metabolic responses [164, 165]. Low temperatures decrease cellular metabolic rates, delay senescence, reduce microbial growth, and can contribute to retaining the quality of fruits after harvest. However, cold storage may also induce several physiological syndromes leading to quality loss, in a condition called CI [57, 168]. CI is a physiological disorder of horticultural products observed in susceptible fruit tissues caused by exposure to temperatures higher than the freezing point, thus differing from freezing damages. CI‐inducing temperatures are variable and dependent on the fruit species; although, in general, susceptible tropical fruit may show symptoms at temperatures lower than 12°C, whereas in more resistant species, the syndrome will manifest itself under 5°C–8°C [168].

Storage temperature and period are considered the most important factors contributing to the appearance of CI in fruit after harvest [57, 168]. In general, CI is caused by chloroplast and mitochondria expansion and disintegration, a decrease in the number and size of starch grains, and accumulation of lipid bodies in chloroplasts and nuclear chromatin [57, 168]. These cellular processes result in a wide range of metabolic disorders, resulting in ripening impairment and flavor and aroma loss [57, 164, 165, 168]. On the outside, CI fruit may exhibit peel abnormalities, such as depression and discoloration, water staining, peel and/or pulp browning, and pulp woodiness or flocculation [169]. Additionally, CI also reduces fruit resistance to microbial pathogens, shortening shelf life after returning to room temperature [57, 168]. Cold‐induced lesions in plum were reduced by exogenous application of methyl jasmonate (MeJA), via modulation of the expression of genes and accumulation of metabolites involved in oxidative homeostasis [170]. Despite its injury‐inducing potential, continuous or intermittent conservation at low temperature remains one of the most important postharvest techniques applied to fruit; in‐depth characterization of specific physiological and metabolic responses using high‐throughput methodologies may contribute to devising the most effective conditions for distinct species. Comprehensive high‐throughput studies have helped to evaluate the effectiveness of the exogenous application of compounds inducing protective pathways before and during cold storage, including melatonin (MT), jasmonates, and salicylic acid (SA) [13–15, 73, 76, 151, 171, 172]. These integrative approaches allow the identification of protective pathways and provide information on their induction, which may contribute to devising novel postharvest conservation technologies.

The differential regulation of genes involved in chromatin structure in response to low temperatures during storage has been demonstrated in apples and other fruit after harvest [24, 57, 173, 174]. Moreover, extensive transcriptional reprogramming has been associated with cold storage [24, 174], suggesting a causal relationship between chromatin remodeling and large‐scale transcriptome modifications. In fact, drastic changes in plant developmental programs have been associated with temperature shifts, such as vernalization, germination induced by stratification, and thermomorphogenesis [78, 156, 175, 176]. Epigenetic events involved in fruit ripening are still a scientific gap. The main changes are known, but there is no development of technological interventions based on this knowledge, especially from a postharvest perspective. These observations hint at the possibility of developing novel conservation technologies capable of inducing large‐scale transcriptional reprogramming via temperature manipulation.

### 3.3. Atmosphere Manipulation

At room temperature, atmosphere composition consists of variable amounts of water vapor, O_2_, CO_2_, N_2_, Ar, and other minor components [177]. The ratio of gaseous components of the atmosphere can be altered under contained environments to prevent fruit senescence after harvest. Technologically designed changes in the relative contents of atmospheric gases around fruits may occur during storage, in the form of controlled atmosphere (CA) storage, or during packaging, as modified atmosphere (MA) [177]. Currently, CA and MA technologies have undergone considerable refinements, giving rise to new approaches, such as dynamic CA and smart MA. These technologies employ sensor‐based analyses of fruit responses to storage conditions coupled with automated adjustments of O_2_ levels during storage according to physiological modifications. Frequently, sensors in dynamic CA and smart MA monitor ethanol production, fruit respiration rates, and chlorophyll fluorescence.

The main biological targets of atmosphere manipulation to preserve fruit after harvest are cellular respiration, redox system activation, and microbial growth impairment [103]. In general, recommended conditions for fruit MA packing consist of O_2_ levels ranging from 1% to 5%, whereas moderate CO_2_ concentrations (10%–20%) are advised for microbial prevention. In contrast, CA and dynamic CA conditions are highly variable and depend on the species [177]. Climacteric pome fruits, like apples, pears, and crab apples, are stored under “double‐low gas” conditions, ranging from 1% to 3% O_2_ and 1% to 3% CO_2_, under low temperatures [177, 178]. In pears, browning can be inhibited and shelf life extended up to 10% O_2_ and CO_2_ [177, 178]. In contrast, drupes, such as peaches, apricots, and dates, are generally stored at O_2_ and CO_2_ concentrations ranging from 3% to 15%, whereas berries are usually stored under single (O_2_) or double (O_2_ and CO_2_) high gases [103, 177]. Compound fruits, including pineapple and dragon fruit, can also be effectively preserved under double‐low gas conditions [177]. The frequency of studies on climacteric and nonclimacteric fruit under CA was similar (Tables 2 and 3), and the biological processes frequently affected by atmosphere manipulation after harvest consist of oxygen and reactive oxygen species (ROS) metabolism, tricarboxylic acid (TCA) cycle, lipid and jasmonic acid pathways, and the *γ*‐aminobutyric acid (GABA) metabolism. In climacteric and nonclimacteric fruit, the interplay between carbohydrate and lipid metabolic pathways is affected under atmosphere manipulation conditions [32, 155, 177].

The key regulator of fruit ripening and senescence, the phytohormone ethylene, naturally occurs in the gaseous form. Thus, several atmosphere manipulation techniques block its action by inhibition, absorption, or oxidation. The inhibitor 1‐methylcyclopropene (1‐MCP), the absorbent zeolite, and catalytic oxidants KMnO_4_, ozone (O_3_), and TiO_2_ can be used to inhibit its action or scavenge the gaseous hormone after harvest [103]. Ethylene scavengers are often used in combination with other atmosphere and temperature manipulation techniques. Among the included studies, the effects of CA on fruit postharvest conservation were most frequently investigated in climacteric fruit (Table 2). Recently, a study with wild type and a nonripening tomato mutant has identified differences in several key genes controlling ripening and demonstrated that the differences in their transcription rate are positively regulated by the expression of their corresponding lncRNAs [179]. The authors demonstrated that a posttranscriptional process, mRNA acetylation, is differentially regulated throughout ripening and in the wild type and mutant. Integrating transcriptome and global mRNA acetylation analyses, the work showed that acetylation has a role in regulating gene expression [179]. The study also demonstrated the differential acetylation of ripening‐related transcripts in the mutant and wild type, suggesting that the differences in ethylene production, fruit texture, and flavor during ripening are controlled by mRNA acetylation [179]. Thus, indicating that posttranscriptional modifications may also function as targets for postharvest conservation techniques.

Other small gaseous molecules, termed gasotransmitters, can be produced endogenously and transmit biological signals, such as hydrogen gas (H_2_), hydrogen sulfide (H_2_S), nitric oxide (NO), carbon monoxide (CO), and methane (CH_4_) [180]. These molecules are produced in response to environmental and developmental conditions and participate in a wide range of processes, including seed germination, root growth, stomatal closure, and responses to abiotic stresses [180]. Exogenous NO application after harvest has been demonstrated to inhibit ethylene biosynthesis, increase antioxidant capacity, induce a stress defense system, activate the C‐repeat binding factor (CBF) pathway, and control sugar and energy metabolism in fruit [181]. The gasotransmitter has several points of interaction with signaling pathways triggered by H_2_S, hydrogen peroxide (H_2_O_2_), oxalic acid (OA), arginine (Arg), GATA factors, or the plant hormone ABA, MT, and MeJA [182]. Similarly, NO has also been demonstrated to influence the expression of genes involved in senescence and to induce protein posttranslational modifications, such as tyrosine nitration, S‐nitrosylation, and nitroalkylation [182–184]. However, its effective use in fruit postharvest conservation is complicated by the short half‐life of gaseous NO and its conversion into nitrogen dioxide (NO_2_), a toxic gas, in the presence of oxygen. The toxic effects of NO_2_ compromise fruit quality by causing tissue death, browning, and discoloration [182]. Therefore, NO fumigation after harvest requires airtight containers to prevent contact with oxygen and N_2_ flushing after NO treatment to prevent NO_2_ damage. The equipment used to generate N_2_ significantly increases production costs. Therefore, NO fumigation to increase fruit postharvest conservation remains restricted to research laboratories [182–184].

Due to its interaction with several hormone pathways and its role in stress responses, H_2_S is considered a gas transmitter of interest for fruit conservation strategies, to enhance fruit quality and prolong shelf life [185]. Its role in alleviating oxidative stress and contributing to preserving cell wall structure has been demonstrated for peaches, tomatoes, and loquat after harvest [13–15, 54, 186, 187]. However, technological aspects concerning the unpredictable kinetics of H_2_S release from donor molecules require further investigation for its use in postharvest applications [185].

### 3.4. Physical Treatments

Recently, physical treatments have emerged as viable, sustainable alternatives to fruit postharvest conservation [188]. These technologies consist of several mechanical and structural approaches employed to manipulate and process horticultural products after harvest. As with other postharvest techniques, physical methods are trifunctional, aiming at quality conservation, shelf‐life extension, microbial contamination, and quality loss reduction [188]. The main advantages of these technologies consist of the replacement of thermal processing and chemical treatment by physical forces, the reduction of nutrient losses in fruits, the enhancement of environmental sustainability, and, consequentially, consumer acceptance. The absence of residues in the treated fruit is also an important advantage of nonthermal physical postharvest treatments [188]. Similarly, transcriptional and metabolic reprogramming has been demonstrated to be less extensive in response to current physical postharvest treatments than under other methods, including low temperature and CA storage [189], although biosynthesis or catalysis of certain metabolites may be influenced by physical treatment of the fruit [188, 190]. The most investigated physical treatments applied to fruit after harvest have been shown to downregulate pathways associated with cell wall modification and induce the metabolism of ROS [190–192]. Gene encoding general signaling components, mitogen‐activated protein kinases (MAPKs), is involved in the regulation of endogenous plant processes, such as growth, development, and programmed cell death, and responses to external conditions, such as temperature shift, water deficit, production of ROS, light, and microorganisms. These genes were also shown to be affected by physical treatment in fruit after harvest [190].

The most prominent nonthermal physical treatments used in postharvest fruit conservation are vacuum‐ and hydrocooling, microwave heating, PEF, CP, high hydrostatic pressure (HHP), UV irradiation and pulsed light, and ionizing radiation [188, 190]. Among the investigated postharvest technologies, physical methods were the most infrequent in omics studies with available data (Tables 2 and 3). The use of physical treatments for conservation after harvest is more commonly reported for vegetables [191, 192].

A study with acerola, a climacteric fruit, demonstrated the effect of pulsed light treatment on several metabolic processes, including cellular respiration, timing of ethylene peak, lipid oxidative metabolism, polyamine, and vitamin C accumulation [193]. The fruit also exhibited increased firmness and reduced weight loss during storage [193]. The authors concluded that treatment with light pulses delayed ripening and promoted quality by activating the antioxidant metabolism. Similar metabolic reprogramming of ascorbic acid accumulation was observed in raspberries and blackberries submitted to cold storage [194]. In blueberries, the integration of physiological and transcriptome data demonstrated that the physical treatment after harvest regulated the dynamic balance of ROS to maintain flesh firmness by promoting the accumulation of compounds with antioxidant activity and the activity of enzymes responsible for ROS scavenging [190]. The authors observed a significant decrease in the contents of H_2_O_2_ in blueberries treated with CP, in comparison to the untreated control. Similarly, the expression of genes encoding cell wall degradation enzymes was reduced in fruit treated with CP [190]. Genes associated with the MAPK signaling pathways were also induced in blueberries submitted to the physical treatment after harvest [190], although it remains unclear whether the observed upregulation is solely caused by the treatment with CP. Studies of fruit treatment with electric field have focused mainly on the inactivation of associated microorganisms, and its role in modulating the kinetics of oxidation reactions remains largely uncharacterized [191, 192]. Electric fields have been demonstrated to affect the contents of nutritionally important metabolites such as small antioxidant molecules and vitamins. The application of electric fields of moderate strength on fruit increases the permeability of cell membranes and has been shown to affect the contents of ascorbic acid and *β*‐carotene in apple slices [195]. In contrast, the application of electric field treatments to vegetables and fruit juice did not affect the content of substances with functional, nutritional, and sensory properties, such as phenolic compounds and vitamins [196]. These contradictory results are likely to be due to the metabolic and cellular differences between vegetables and fleshy fruit. The effects of physical aspects of electric fields applied to fruit conservation, including the strength of the electric field, its frequency, pulse width, total treatment time, and specific energy, are scarcely characterized, and high‐throughput integrative studies may contribute to establishing effective conditions for obtaining safe and stable products. Studies on postharvest conservation technologies based on physical treatments remain restricted to certain berries, and integrative omics studies are still scarce (Tables 2 and 3).

### 3.5. Chemical Treatments

Traditionally, synthetic chemical products, such as chlorine dioxide, NO, SA, 1‐MCP, and several insecticides and fungicides, are used in fruit preservation to extend shelf life and maintain the quality after harvest [197]. However, most recently, consumer preferences have driven the use of biological products in the chemical treatment of fruit after harvest, including biological control agents and plant‐based products [197]. The main biological target of fruit chemical treatment is the inhibition of microbial growth, along with desiccation protection after harvest and during storage. Accordingly, the food industry has increased the use of bioactive compounds with antioxidant and antimicrobial activity. Biological products can be effective replacements for synthetic compounds in fruit preservation after harvest and during storage. Natural elicitors, such as phenylalanine, have been demonstrated to activate defense‐related pathways in fruit, including lipoxygenases and phenylpropanoid biosynthesis [198]. Edible packaging is considered an alternative to the use of chemical products and biocidal agents in the conservation of fruit after harvest [197, 199]. A wide range of biopolymer molecules, including polysaccharides, proteins, lipids, waxes, essential oils, and nanoparticles, have been used in fruit postharvest applications [197, 199]. Moreover, products of plant specialized metabolism, such as polyphenols and phenolic acids, terpenoids and other volatiles, and aldehydes and complex plant extracts, along with organic compounds of microbial and animal origins, have also been used in conservation strategies for fruit after harvest [197, 199]. In horticultural products, including fruit, antifungal activity against *Aspergillus niger*, *Penicillium digitatum*, *Penicillium italicum*, *Botrytis cinerea*, and several species of *Fusarium* has been demonstrated for essential oil terpenoids, such as linalool, citral, citronellal, *α*‐terpineol, carvacrol, eugenol, octanal, and thymol, plant aldehydes perill‐ and cuminaldehyde, alkaloids, saponins, tannins, and polyphenols, including anthocyanins, cinnamic acid, and tannic acid [197]. Moreover, complex metabolite mixtures found in plant extracts were also used to prevent microbial growth in horticultural products, including postharvest fruit [197, 199], including domesticated species, such as garlic, neem, mint, basil, and thyme leaf extracts, and extracts from several parts of wild species, including *Anvillea radiata*, *Asteriscus graveolens*, *Bubonium odorum*, *Ceratonia siliqua*, several *Cistus* species, *Hammada scoparia*, *Ighermia pinifolia*, *Inula viscosa*, *Halimium umbellatum*, *Rubus ulmifolius*, and *Sanguisorba minor* ([197, 200]. However, acute ingestion of essential oils has been demonstrated to trigger severe allergic reactions, damage mucous membranes, promote deterioration of the liver, and reduce the levels of glucose in the blood serum, which may lead to convulsions and coma [201]. In agricultural products not destined for in natura consumption, such as grains, plant‐derived essential oils are considered promising alternatives to synthetic biocides during storage [202]. In the case of fruit, destined for human consumption with no or minor processing, the use of biological agents and products in postharvest conservation requires thorough investigation of their many potential mechanisms of action, associated with possibly distinct efficacy and side effects [164, 165, 203]. Ideally, a biological control product should be reliable, effective, widely accepted, patent protected, registered, and suitable for commercialization [164, 165]. The number of products attaining the desired qualities remains small, and the available biocontrol products represent a minor portion of the market [164, 165]. Recently, an edible fruit coating developed by Apeel Sciences has been proposed for organic fruit, consisting of plant‐based monoglycerides and diglycerides, citric acid, and sodium bicarbonate [204]. However, the product has been discontinued in 2023, likely due to regulatory gaps concerning its composition and mechanism of action [205]. The situation reinforces the need for a stronger scientific basis for the recommendation of fruit coating products, especially for organic systems.

Similarly, the representativeness of integrative, large‐scale studies on fruit after harvest remains scarce. Biocontrol strategies have evolved into combinatory approaches using several microbial antagonists or the combination of microbes with physical and chemical techniques [164, 165, 203]. Alternatively, the use of physical agents before storage, including far‐red light and ultraviolet radiation, has been demonstrated to reduce cold‐induced damages in tomato [206]. Exogenous applications of salicylates, jasmonates, and MT (*N*‐acetyl‐5‐methoxytryptamine) were also effective in increasing the levels of intracellular energy, enhancing the activity of Cytochrome c oxidase enzymes, and preserving membrane fluidity and integrity in tomato [207]. In apple, treatment of harvested fruit with exogenous MT also impaired ethylene production and delayed ripening [208]. The role of MT as a suppressor of ripening was further confirmed by the inverse correlation between the contents of endogenous MT and ethylene production [208]. The compound also repressed the transcription of key genes in ethylene biosynthesis, such as *MdACS1* (*1-AMINOCYCLOPROPANE-1-CARBOXYLIC ACID SYNTHASE*) and *MdACO1* (*ACC OXIDASE*), during ripening. Similarly, exogenous MT treatment reduced the expression of transcription factors *MdREM10* (*REPRODUCTIVE MERISTEM10*) and *MdZF32* (*ZINC FINGER32*). The protein *Md*REM10 was shown to bind to the promoter of *MdERF3* (ET*HYLENE RESPONSE FACTOR3*), inducing its transcription, which in turn promoted the transcription of *MdACS1* [208]. The work also demonstrated that *Md*REM10 directly binds the promoter of *MdZF32*, whose active protein binds the promoter of the *MdACO1* promoter, inducing its expression and closing the regulatory loop. The findings demonstrate the potential of MT in apple postharvest conservation. However, information on other fruit species remains largely unavailable. Integrated large‐scale approaches may contribute to expanding the current knowledge on the effect of physical agents on less characterized tropical fruit, allowing the development of novel conservation technologies. A simultaneous increase in the number of omics studies on the effects of biological control agents in fruit after harvest would help provide invaluable insight into fruit processes leading to improved quality or extended storage.

### 3.6. Biological Processes Affected by Postharvest Technologies

After harvest, fruits undergo metabolic and physiological changes, including alterations in pulp firmness, specialized metabolism that controls color and flavor, changes in nutritional content, and macromolecular degradation processes affecting proteins, lipids, and nucleic acids [209]. Although common pathways are shared, the senescence of fruit attached to the plant also involves different processes [9, 210]. Studies of ripening and senescence of fruit attached to the plant were not included in the current review. In this study, initial meta‐analyses were conducted separately for climacteric and nonclimacteric fruits to investigate if common pathways could be detected among the different species within the two ripening patterns (Figures 1 and 3a). Subsequently, to achieve a more complete overview of fruit developmental and metabolic processes after harvest, the analyses integrated data from climacteric and nonclimacteric fruits (Figures 3b and 4). To integrate information from distinct omics studies and fruit species, we employed GO terms, which consist of a dynamic and controlled vocabulary that allows classification of biological molecules into expanding and changing categories of biological processes, molecular functions, and cellular components. The analyses focused on terms referring to biological processes since they were designed to describe larger biological phenomena, consisting of a series of events brought about by the ordered assembly of molecular functions occurring in one or more cellular compartments. The use of GO terms allows the comparison of processes described inconsistently in different studies, thus providing a unifying nomenclature for different studies. Omics studies in postharvest fruit ripening provide novel insights into the complex physiological and biochemical processes and regulatory mechanisms responsible for developmental changes. Moreover, integrated omics allow simultaneous investigations of several steps of the transmission of genetic information, from basic genetic information contained in the DNA to effector molecules acting on the metabolism. Comprehension of the molecular mechanisms underlying physiological changes in fruit after harvest and during storage, provided by integrative studies, may contribute to the development of sustainable postharvest strategies to increase quality, impair senescence, and extend shelf life.

Figure 3Scatterplot of GO enrichment analyses in (a) climacteric and (b) nonclimacteric fruit omics studies. The number of studies is represented by sphere size (count) and GO enrichment by log10 *p* value. (c) Multivariate sparse partial least square (sPLS) model of GO biological process terms in climacteric and nonclimacteric fruits using species as discriminant (DA).

**Figure:**
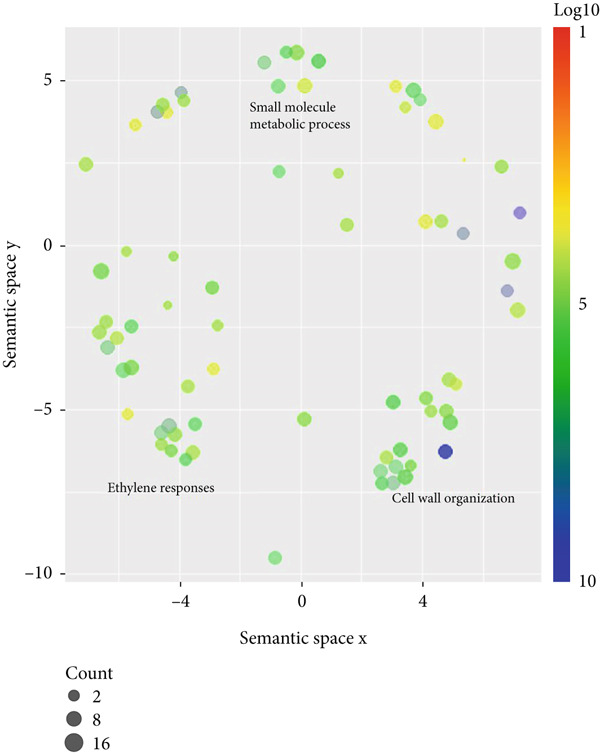
(a)

**Figure:**
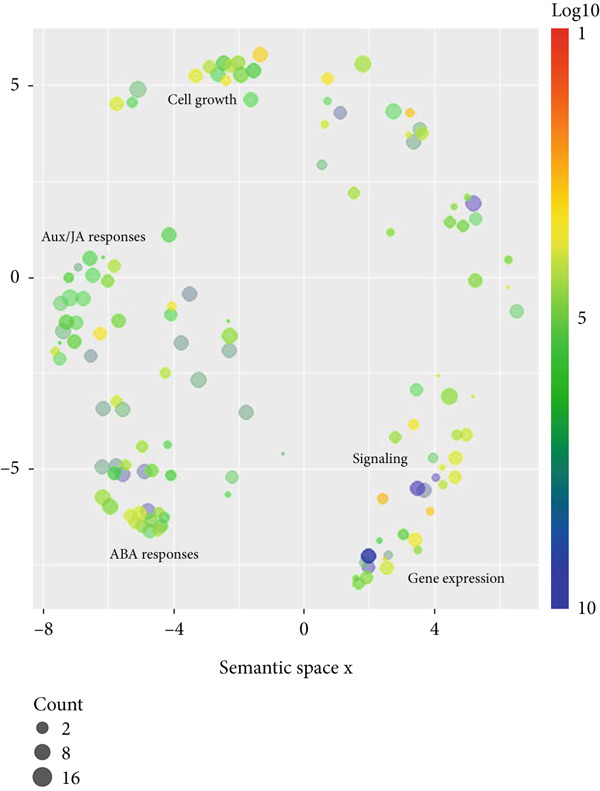
(b)

**Figure:**
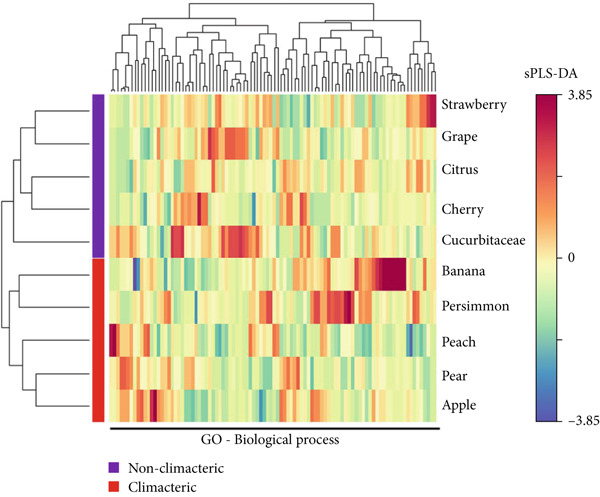
(c)

Figure 4(a) Heatmap representation and cluster analysis of the normalized frequency of Gene Ontology (GO) terms in the omics studies in association with the investigated outcomes. (b) Relevance network analysis of hierarchically superior GO groups (biological process) and postharvest outcomes. Significant associations at cutoff = 0.75 are shown. (c) Association between GO terms from the omics studies and the conservation technologies represented as risk ratio at 95% confidence interval (CI).

**Figure:**
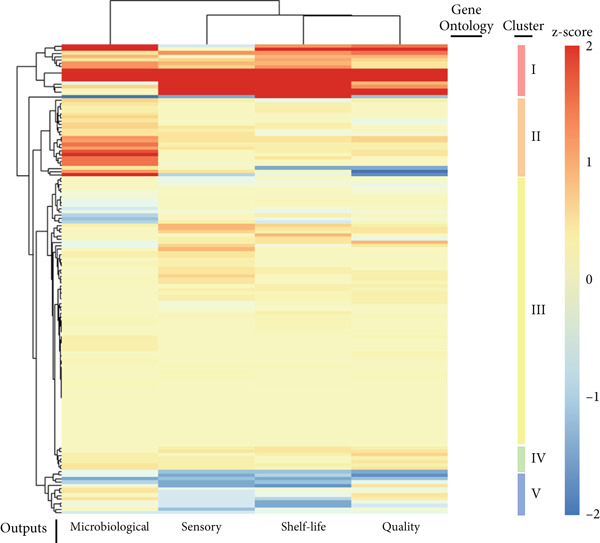
(a)

**Figure:**
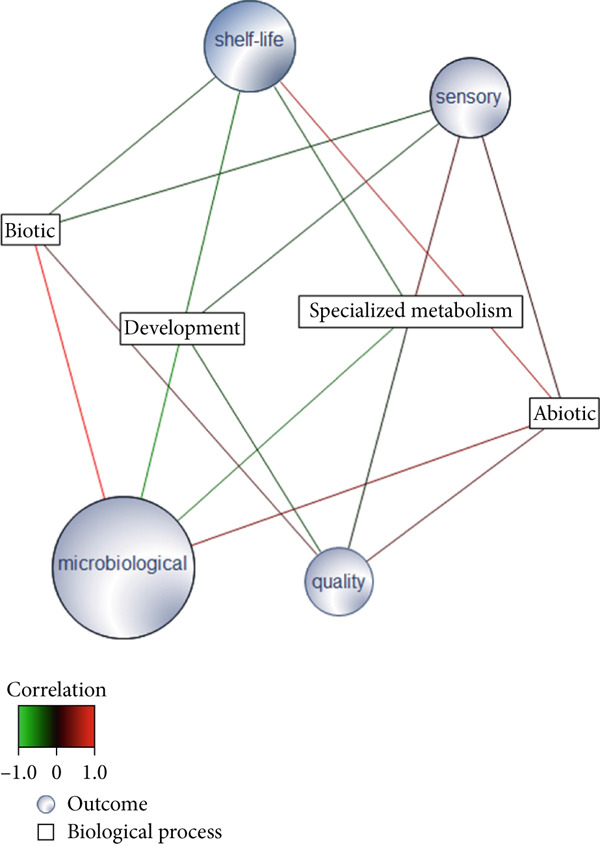
(b)

**Figure:**
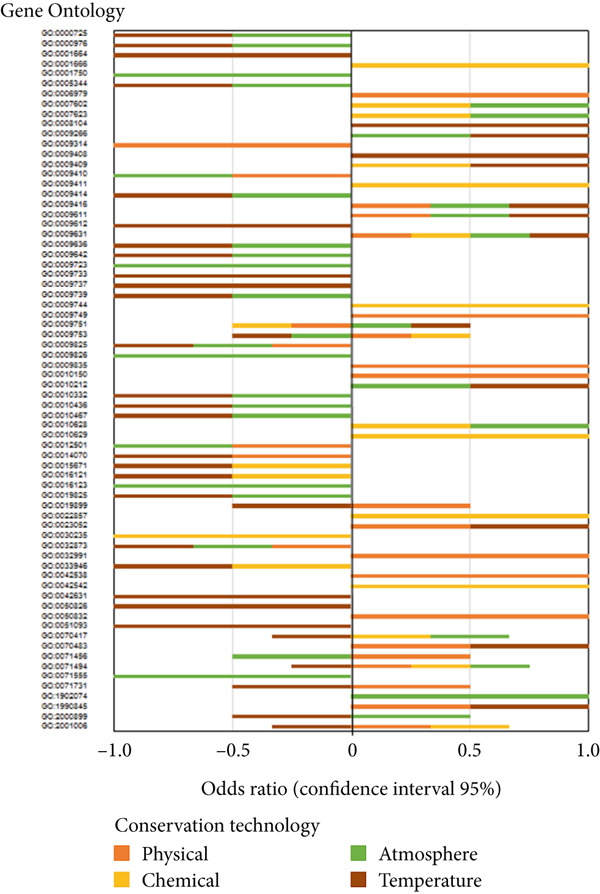
(c)

Meta‐analyses of GO categories from the studies demonstrate the prevalent role of ethylene in climacteric fruit after harvest (Figure 3a). Endogenous ethylene production, controlled by an autoregulatory feedback loop, displays significant interaction with signaling pathways mediated by other hormones, such as ABA, JA, and auxin. Accordingly, GO terms associated with responses to most hormones were significantly enriched in the meta‐analyses of the results from studies with climacteric fruit (Figure 3a). The impact of postharvest technologies is represented by the enrichment of terms associated with defense processes, including responses to toxic substances and defense against fungus (Figures 3a and 4c), along with the shared terms fruit ripening, cell wall organization, and small molecule metabolic process. In contrast, in nonclimacteric fruit, processes associated with anabolic processes, such as auxin and ABA responses, signaling, and cell growth, remain significant after harvest (Figure 3b). Terms associated with conservation techniques include responses to light, reactive oxygen metabolic processes, regulation of protein serine/threonine kinase activity, and pectin metabolic process (Figures 3b and 4c). These results agree with the current knowledge on fruit ripening and senescence [8, 78, 96, 156, 211, 212] and demonstrate the validity of the integrative approach using GO terms used in the meta‐analyses. The integration of information from several regulatory layers controlling fruit postharvest may guide the development of specific and effective technologies, such as the targeted use of ethylene inhibitors, hormone regulators, and physical or chemical agents adapted to the fruit type and its physiological profile. Results from integrative approaches allow the identification of biomarkers associated with senescence and quality loss that can be incorporated in real‐time monitoring systems throughout the storage and commercialization chain.

Processes controlled by other hormones were not significantly represented in the meta‐analyses for climacteric fruit, whereas GO representing processes controlling small molecule metabolism was detected (Figure 3a). As expected, GO meta‐analyses highlighted the close interaction of growth with ripening and senescence in nonclimacteric fruit, providing further evidence of the association between developmental processes and exogenous environmental responses (Figure 3b). The integration of data from climacteric and nonclimacteric omics studies retained a defined separation between the two categories of ripening (Figure 3c); however, the intermediate patterns observed in certain fruit, such as melon [213] in the Cucurbitaceae family, were confirmed (Figure 3c). Similarly, integrative meta‐analyses highlighted the divergent ripening and postharvest behavior of model climacteric and nonclimacteric fruits, apple and strawberry, respectively (Figure 3c).

High‐throughput studies also allow investigations of processes occurring in distinct fruit tissues. Multiomics analyses demonstrated significant differences in ripening regulation between the pulp and peel of banana after harvest [214]. In the study, peel ripening was significantly controlled by genes associated with transcriptional regulation, hormone signaling, cell wall modification, protein modification, and energy metabolism [214]. In the pulp, genes classified in transcriptional regulation, signal transduction, and cell wall modification ontologies were also significantly induced, along with secondary metabolism GO, which was not altered in the peel [214] (Tables 2 and 3). In contrast, at the protein level, energy metabolism, oxidation–reduction reactions, cell wall metabolism, and starch degradation were most significantly altered [214] (Table 2). Accordingly, secondary metabolism, energy metabolism, and protein metabolic processes were found to be involved in banana pulp ripening [215, 216].

Multivariate modeling of the GO terms using the investigated outputs as discriminants clustered the biological processes in five distinct groups (Figure 4a). The first cluster (I) is consistently overrepresented postharvest technologies contributing to all investigated outputs (Figure 4a) and consists of DNA binding, gene expression, signaling, and intracellular processes. Thus, it reinforces the role of conservation techniques in reprogramming fruit development. The second group of GO (II), more closely associated with microbiological conservation, consists of pathogenesis‐related (PR) responses, biotic agents, and UV light processes (Figure 4a). The ontologies in the third cluster (III) represent general ripening processes, such as responses to hormone, responses to ethylene, responses to ABA, carbohydrate metabolic processes, and specialized metabolism (Figure 4a). The smallest group of GO (IV) is negatively associated with sensory and shelf‐life outcomes, corresponding to catabolic processes, hydrolytic activity, and senescence (Figure 4a). The last group of GO, also negatively associated with sensory, quality, and shelf‐life outcomes, consists of general catabolic activity, metabolite, and cellular component degradation (Figure 4a). A relevance network constructed based on the sPLS‐DA model highlighted the positive association of developmental GOs with shelf life, sensory, and quality outcomes (Figure 4b). In contrast, GO terms corresponding to responses to biotic factors are significantly associated with microbiological conservation (Figure 4b), and those referring to specialized metabolism have a negative association with microbiological and shelf life, but a neutral significant association with sensory and quality outcomes (Figure 4b). These apparently contradictory observations may be explained by the complex role of specialized metabolism in plant protection and evolution of sensory properties [217, 218]. Specialized metabolites have been demonstrated to be synthesized in plants in response to several biotic and abiotic stresses [217, 218], providing effective protection against insects, herbivores, and pathogens and contributing to mitigate the deleterious effects of environmental factors, such as ultraviolet radiation and extreme temperatures [218]. In fruit during ripening and postharvest conservation, intermediate metabolites produced by the central carbon metabolism function as precursor molecules for several pathways in the specialized metabolism [218]. Compounds produced by specialized metabolism are important contributors to fruit sensory properties during ripening, such as flavor and aroma [217, 218]. However, conservation technologies have been demonstrated to exert distinct effects on primary and specialized metabolism [9, 24, 212].

Microbial growth and colonization of fruit after harvest cause visual depreciating symptoms, including mold, rot, discoloration, softening, shriveling, browning, or blackening [94]. The most common fungal pathogens affecting fruit postharvest include *B. cinerea*, *Penicillium* spp., and *Alternaria* spp. [219]. Elicitor compounds, biocontrol agents, and genetic approaches are innovative techniques generally recognized as safe to inhibit microbial growth on fruit after harvest [54, 187]. Comprehensive omics studies may contribute to elucidating the molecular components responsible for the complex interactions among fruit microbial populations after harvest and during storage. In banana peel, postharvest application of exogenous MT delayed anthracnose (*Colletotrichum musae*) pathogenesis by modulating the activity of receptor kinases associated with auxin, ethylene, and MAPK pathways [215, 216] (Table 2). Similarly, the expression of genes responsible for cell wall and wax metabolism was induced by MT application [215, 216] (Table 2). In mandarin citruses, naringenin reduced the onset of microbial colonization by inducing the accumulation of metabolites with antimicrobial activity, such as auraptene, butin, naringenin, and luteolin [220] (Table 3). The metabolic reprogramming was accompanied by increased expression of genes associated with fruit specialized metabolism, including *CcPGT* (phlorizin synthase), *CcFNS* (flavone synthase), *CcF3H* (flavanone 3‐hydroxylase), *CcF3′H* (flavonoid 3′‐hydroxylase), *CcFLS* (flavonol synthase), and *CcUGT*s (UDP‐glycosyltransferase) in the flavonoid and phenylpropanoid biosynthesis pathways, promoting tolerance against pathogenic infection [220].

In grape, tolerance to *B. cinerea* was induced by postharvest application of chitosan via transcriptional activation of genes involved in disease perception, plant hormone biosynthesis, signal transduction, and secondary metabolism [183, 184]. In contrast, ethanol applications to fruit after harvest significantly repressed the expression of disease resistance–related protein families, including PR proteins and chitinase, leading to reduced accumulation of SA‐mediated defense pathways [221] (Tables 2 and 3).

The use of biological control yeast *Yarrowia lipolytica* to prevent blue mold (*Penicillium expansum*) in apples after harvest promotes the accumulation of PR proteins and induces the transcription of defense genes [25] (Table 2). Integrative transcriptome and proteome studies demonstrated that defense gene and protein induction were mediated by SA, jasmonate, and ethylene signal transduction pathways [25] (Table 2). Oxidative stress and PR proteins were also induced by the biological control yeast [25]. Similar transcriptional and proteomic responses, including the upregulation of disease resistance genes and jasmonate‐responsive transcription factors, were observed in pears receiving treatment with postharvest biocontrol agent *Wickerhamomyces anomalus* [183, 184]. In contrast, in grapes and citrus treated with *Y. lipolytica* after harvest, genes and proteins associated with stress responses, energy production, signal transduction, and oxidoreductase activity were upregulated [222] (Table 3). Integrated transcriptome and proteome analyses of strawberries treated with biocontrol agent *Rhodotorula mucilaginosa* and chitosan after harvest have shown extensive activation of the jasmonate, ethylene, ABA, and gibberellic acid signaling pathways leading to enhanced transcription of disease resistance genes [223] (Table 3). Hormone‐mediated pathways also led to the induction of several gene encoding enzymes controlling the biosynthesis of resistance compounds, such as BAHD acyltransferase, vinorine synthase, UDP‐glycosyltransferase, flavonol synthase, and long‐chain acyl‐CoA synthetase [223] (Table 3).

Brassinolide treatment has been demonstrated to alleviate symptoms of CI, and multiomics studies have shown that the steroid hormone precursor regulates the lipoxygenase activity in the *α*‐linolenic acid pathway, enhancing jasmonic acid–CoA (JA‐309 CoA) synthesis, which prevents cell wall and membrane lipid damage [224]. Similarly, postharvest application of MeJA reduces CI by inducing the transcription of genes encoding key enzymes of plant hormone, antioxidant, phospholipid, and cell wall modification pathways [224]. Proteins involved in glutathione and fatty acid metabolism were also associated with MeJA‐mediated alleviation of CI symptoms in fruit [128] (Tables 2 and 3). Exogenous MT applications were shown to repress the transcription of MYB factors controlling cell wall and energy supply metabolism in cold‐stored fruit [225].

The most representative GO terms corresponding to biological processes in the work selected for the meta‐analyses include recombinational repair, transcription *cis*‐regulatory region binding, G protein–coupled receptor binding, response to hypoxia, photoreceptor outer segment, oxygen carrier activity, response to oxidative stress, phototransduction, circadian rhythm, protein localization, response to temperature stimulus, response to radiation, response to heat, response to cold, response to xenobiotic stimulus, response to UV, response to water deprivation, response to light stimulus, response to wounding, response to mechanical stimulus, cold acclimation, response to toxic substance, response to light intensity, response to ethylene, response to auxin, response to ABA, response to gibberellin, response to sucrose, response to glucose, response to SA, response to jasmonic acid, multidimensional cell growth, unidimensional cell growth, fruit ripening, leaf senescence, response to ionizing radiation, response to gamma radiation, carotenoid dioxygenase activity, gene expression, positive regulation of gene expression, negative regulation of gene expression, programmed cell death, response to organic cyclic compound, oxygen transport, carotene catabolic process, xanthophyll biosynthetic process, oxygen binding, enzyme binding, transmembrane transporter activity, signaling, NO synthase regulator activity, negative regulation of stress‐activated MAPK cascade, protein‐containing complex, xyloglucan‐specific endo‐beta‐1,4‐glucanase activity, hyperosmotic salinity response, response to H_2_O_2_, cellular response to water deprivation, response to freezing defense, response to fungus, negative regulation of developmental process, cellular response to cold, detection of hypoxia, cellular response to hypoxia, cellular response to UV‐C, cell wall organization, response to NO, response to salt, adaptive thermogenesis, xyloglucan catabolic process, and regulation of cellulose biosynthetic process (Figure 4c, Table S1, Table S2).

The OR of the most representative GO terms in the studies included in the meta‐analyses reinforced the role of temperature and atmosphere manipulation to attain the outcomes (Figure 4c). These results contribute to demonstrating the potential of epigenetic changes, induced by low temperatures, in regulating large transcriptional programs in fruit and extending conservation, or even promoting metabolic reprogramming, as shown in apple [24]. The control of chromatin structure and, consequently, gene expression activity is dependent on reversible chemical modifications in the DNA and histone proteins constituting nucleosomes. Methylation of DNA, coding and noncoding RNA, and histone posttranslational modifications, such as acetylation, SUMOylation and ubiquitination, methylation, and phosphorylation, were shown to affect ripening and senescence in apple, apricot, banana, kiwifruit, sweet cherry, and peach [45–47, 79, 226–230]. Epigenetic changes are frequently induced by temperature shifts [78, 156, 176], indicating the potential of temperature manipulation in genetic reprogramming to improve fruit conservation after harvest. However, the transient nature of epigenetic modifications may pose additional difficulties in the commercialization steps after storage.

Although epigenetic changes have been demonstrated to induce partial metabolic reprogramming in fruit [24, 45–47, 79, 226–230], subsequent senescence is considered largely irreversible [8]. In fleshy fruit model species tomato, ultraviolet C has been demonstrated to delay ripening and retain fruit quality after harvest by chromatin remodeling and inducing methylation of ethylene‐associated genes [231]. In citrus and tomato, a combination between exogenous hormone application (GA and a synthetic cytokinin, 2‐isopentenyladenine) and additional fluorescent light to natural sunlight background has been shown to re‐establish chlorophyll biosynthesis, promote chloroplast redifferentiation, and induce regreening after harvest [232]. The induction of these juvenile traits did not affect other senescence‐related processes, such as firmness or weight loss [232]. Thus, although several postharvest approaches may delay some senescence processes, they remain highly species‐specific and restricted to certain metabolic pathways.

**Figure 5:**
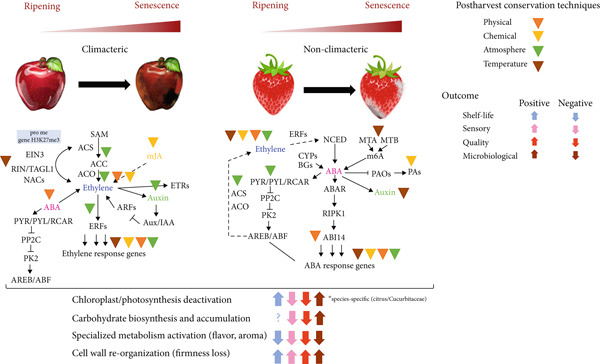
Schematic representation of the molecular components involved in postharvest ripening and senescence in climacteric and nonclimacteric species, the targets of conservation technologies, and their effects on the outcomes investigated in the review. Involved hormones are represented as ethylene, abscisic acid (ABA), auxin, and methyl jasmonate (MeJA). Dashed lines represent hypothetical interactions. Induction is shown as an arrowhead and repression as a horizontal line. Metabolites correspond to 1‐amino‐cyclopropane‐1‐carboxylic acid (ACC), polyamines (PAs), and S‐adenosyl‐l‐methionine (SAM). Proteins involved in metabolic and signaling pathways are shown as ACC synthase (ACS), ACC oxidase (ACO), ABA‐response element binding factor/ABA‐responsive factor (AREB/ABF), ABA receptor (ABAR), related to ABA Receptor 14 (AB14), auxin response factors (ARFs), auxin/indole‐3‐acetic acid transcription modulators (Aux/IAA), *β*‐glucosidases (BGs), cytochrome P450 (CYPs), ethylene‐insensitive 3 (EIN3), ethylene responsive genes (ETRs), Methyltransferase A (MTA), Methyltransferase B (MATB), NAM, ATAF1/2, and CUC2 family (NAC), 9‐*cis*‐epoxycarotenoid dioxygenases (NCEDs), polyamine oxidase (PAO), pyrabactin resistance (PYR)/PYR‐like (PYL), Type 2C protein phosphatase (PP2C), Protein Kinase 2 (PK2), ripening inhibitor (RIN), regulatory components of ABA receptor (RCAR), receptor‐interacting protein kinase (RIPK), and tomato AGAMOUS‐LIKE 1 (TAGL1). Epigenetic modifications are represented as promoter region methylation (pro me), gene body histone methylation (gene H3K27me3), and N6‐methyladenosine methylation (m6A). The targets of postharvest technologies are shown as color‐coded triangles, and the outcome of the conservation techniques is represented by arrows.

The current analyses reinforce the biological function of enzymatic kinetics in fruit processes after harvest, for desirable outcomes as development of sensory properties and as a deleterious factor in shelf‐life period and microbiological conservation (Figure 4c). The main genes and pathways affected by the conservation technologies investigated in climacteric and nonclimacteric fruit are summarized in Figure 5. Although useful to summarize the meta‐analyses results, the OR is a simplified representation of the complex interactions between process and environmental factors controlling fruit ripening and senescence after harvest. Therefore, we consider that models constructed from large data analyses are a better representation of the influence of development and metabolism on the investigated outcomes.

## 4. Strengths and Weaknesses

To the best of our knowledge, this is the first systematic review and meta‐analysis of omics data of fruit postharvest conservation focusing on four outputs: sensory, quality, shelf life, and microbiological aspects. Other reviews have focused on stress and defense responses after harvest [233, 234] or on the control of ripening, including in planta studies [235]. Recently, a review work has investigated the molecular mechanisms underlying postharvest physiology and metabolism of fruit in multiomics studies [210]; however, it does not consider specific outcomes and did not perform meta‐analyses of the published data. In our work, a comprehensive search strategy was used to mine five scientific databases and a list of relevant publications, and their primary data were submitted to meta‐analysis. The exclusion of studies where biases were detected helped to ensure an evidence‐based meta‐analysis. However, the integration of data from distinct fruit species is aimed at providing a general overview of the biological processes affected during conservation, without attributing differential weights to specific outcomes that may be distinctly sought after in different fruits. For example, challenges for the conservation of highly perishable fruit such as grapes and bananas are different from those necessary to expand and improve the quality of pears and apples that can remain for more than 9 months under storage conditions after harvest. Thus, the general panorama drawn by the current analyses does not override individual requirements faced by particular fruit species. Instead, the integrated analyses are aimed at identifying crucial pathways associated with the desired outcomes that remain relatively unexplored in the development of conservation technologies.

The weaknesses of the work are the restricted geographic location of the included work and the elimination of studies written in languages other than English. Thus, potentially relevant data may have been kept out of the analyses. Moreover, distinct biological constraints of the investigated fruit, leading to different study designs, may have affected the meta‐analyses′ outcome. In addition, a bias toward studies with pomes and berries may not have been completely neutralized by the normalization techniques applied.

## 5. Conclusion

The current systematic review and meta‐analysis confirm the crucial role of postharvest technologies in contributing to fresh fruit availability, nutrition, and food security worldwide. The potential of high‐throughput, integrative studies in unveiling novel targets to develop conservation strategies is also presented, although large‐scale studies on biocontrol agents after harvest and combined approaches remain scarce. The systematic review also highlights the gap between basic knowledge and technological applications to improve fruit postharvest conservation. When we analyzed the level of use of scientific knowledge generated in the articles included in the meta‐analyses, the application of cold storage and atmosphere control technologies, including modified, controlled, and dynamic atmosphere, and the use of chemical compounds and physical agents are in full use and consolidated, with TRLs between 8 and 9. The remaining postharvest technologies remain largely restricted to experimental studies. Furthermore, a restricted set of fruit constitutes the group most benefiting from these technologies, such as apples, pears, mangoes, peaches, grapes, strawberries, and citrus. In the case of articles whose central theme is omics and their potential applications, TRL remains medium‐low (three to five), restricted to laboratory level or as a research tool to monitor consolidated technologies. Thus, here lies a big challenge for future research: to transform the scientific knowledge from omics studies into technological products. The compiled results demonstrate that these advances are occurring faster in preharvest than in postharvest.

Recent findings on the function of epigenetic components in controlling plant development in response to environmental cues, such as temperature, may provide novel alternatives in the development of conservation technologies for fruit after harvest. Our meta‐analyses reinforce the crucial function of phytohormones in climacteric and nonclimacteric fruit conservation, although demonstrating a closer integration between ripening and senescence in nonclimacteric fruit. Ethylene‐ and ABA‐controlled processes are the main contributors to senescence, negatively affecting all investigated outcomes in climacteric and nonclimacteric fruits, respectively. Gene ontologies associated with carbohydrate and reactive oxygen metabolic pathways were shown to be important players in conservation strategies. Epigenetic modifications, as DNA methylation and histones posttranslational changes, are promising targets for novel conservation techniques. Our review and analyses indicate that further research on epigenetic factors on fruit genomic, transcriptional, and metabolic changes may contribute to devising novel, paradigm‐changing postharvest alternatives.

## Conflicts of Interest

The authors declare no conflicts of interest.

## Author Contributions

Tatiane Timm Storch: conceptualization, methodology, formal analysis, investigation, writing—original draft, and visualization; Camila Pegoraro: conceptualization, methodology, formal analysis, investigation, writing—original draft, and visualization; Vera Quecini: conceptualization, methodology, formal analysis, investigation, writing—review and editing, and visualization; Cesar V. Rombaldi: conceptualization, writing—review and editing, and supervision; César L. Girardi: conceptualization, writing—review and editing, and supervision.

## Funding

This study was funded by the Conselho Nacional de Desenvolvimento Científico e Tecnológico (306771/2014‐4 and 441856/2014‐4), the Empresa Brasileira de Pesquisa Agropecuária (02.13.05.014.00.00), the Coordenação de Aperfeiçoamento de Pessoal de Nível Superior (15/2014), and the Comité Français d′Évaluation de la Coopération Universitaire et Scientifique avec le Brésil (631/09).

## Supporting information


**Supporting Information** Additional supporting information can be found online in the Supporting Information section. Table S1: Gene Ontology (GO) biological processes significantly affected by postharvest treatments and the investigated outputs in climacteric fruit in the articles included in the meta‐analyses. Table S2: Gene Ontology (GO) biological processes significantly affected by postharvest treatments and the investigated outputs in nonclimacteric fruit in the articles included in the meta‐analyses. Table S3: Odds ratio of Gene Ontology (GO) biological processes significantly affected by postharvest treatments and the investigated outputs at *p* < 0.05. Postharvest treatments were grouped in physical treatments, atmosphere manipulation, temperature manipulation, and edible coating. Table S4: Quality assessment appraising relevance, reliability, validity, and applicability of the evidence and risk of bias of the articles included in the meta‐analyses. Figure S1: Risk‐of‐bias assessment of the omics studies included in the systematic review. Individual and overall domains are represented by bars and risk by colors.

## Data Availability

Data is provided as supporting information.
