# Supplementary material for: Novel Targets for Fruit Conservation Strategies Revealed by Omics Studies: A Systematic Review and Meta‐Analysis
Source: Int J Food Sci. 2025 Oct 29;2025:9963581. doi: 10.1155/ijfo/9963581 (PMC12569611; doi:10.1155/ijfo/9963581)
Supplement: Supplementary file 1 — Supporting Information Additional supporting information can be found online in the Supporting Information section. Table S1: Gene Ontology (GO) biological processes significantly affected by postharvest treatments and the investigated outputs in climacteric fruit in the articles included in the meta‐analyses. Table S2: Gene Ontology (GO) biological processes significantly affected by postharvest treatments and the investigated outputs in nonclimacteric fruit in the articles included in the meta‐analyses. Table S3: Odds ratio of Gene Ontology (GO) biological processes significantly affected by postharvest treatments and the investigated outputs at p < 0.05. Postharvest treatments were grouped in physical treatments, atmosphere manipulation, temperature manipulation, and edible coating. Table S4: Quality assessment appraising relevance, reliability, validity, and applicability of the evidence and risk of bias of the articles included in the meta‐analyses. Figure S1: Risk‐of‐bias assessment of the omics studies included in the systematic review. Individual and overall domains are represented by bars and risk by colors. [file IJFO-2025-9963581-s001.zip › SupplementaryMaterial.docx]

**Supplementary Material**

**Supplementary Tables**

**Table S1**. Gene Ontology (GO) Biological Processes significantly affected by postharvest treatments and the investigated outputs in climacteric fruit in the articles included in the meta-analyses.

**Table S2**. Gene Ontology (GO) Biological Processes significantly affected by postharvest treatments and the investigated outputs in non-climacteric fruit in the articles included in the meta-analyses.

**Table S3**. Odds ratio of Gene Ontology (GO) Biological Processes significantly affected by postharvest treatments and the investigated outputs at p < 0.05. Postharvest treatments were grouped in physical treatments, atmosphere manipulation, temperature manipulation, and edible coating.

**Table S4**. Quality assessment appraising relevance, reliability, validity, and applicability of the evidence and risk-of-bias of the articles included in the meta-analyses.

**Supplementary Figure Caption**

**Figure S1**. Risk of bias assessment of the omics studies included in the systematic review. Individual and overall domains are represented by bars and risk, by colors.

**Supplementary Tables**

**Table S3**. Odds ratio of Gene Ontology (GO) Biological Processes significantly affected by postharvest treatments and the investigated outputs at p < 0.05. Postharvest treatments were grouped in physical treatments, atmosphere manipulation, temperature manipulation, and edible coating.

| **GO** | **BiologicalProcess** | **sensory** | **shelf_life** | **microbiological** | **quality** |
| --- | --- | --- | --- | --- | --- |
| GO:0000325 | plant-type_vacuole | 0.05 | 0 | 0 | 0.05 |
| GO:0000976 | transcription_cis-regulatory_region_binding | 0.5 | 0 | 1.25 | -2 |
| GO:0001223 | transcription_coactivator_binding | 0 | -1.5 | 0.5 | -1.5 |
| GO:0001666 | response_to_hypoxia | 2 | 2 | -0.5 | 1 |
| GO:0001786 | phosphatidylserine_binding | 0 | 0.25 | -1 | 0 |
| GO:0003700 | DNA-binding_transcription_factor_activity | -0.75 | 1.25 | 2 | 1.5 |
| GO:0003724 | RNA_helicase_activity | 0 | 0 | 0 | 0 |
| GO:0005509 | calcium_ion_binding | 2 | 2 | 0 | 2 |
| GO:0005515 | protein_binding | 0 | 0 | 0.5 | -0.5 |
| GO:0005544 | calcium-dependent_phospholipid_binding | 2 | 2 | 0 | 2 |
| GO:0006816 | calcium_ion_transport | 2 | 2 | 2 | 2 |
| GO:0006970 | response_to_osmotic_stress | 2 | 2 | 2 | 2 |
| GO:0006979 | response_to_oxidative_stress | 2 | 2 | 2 | 2 |
| GO:0007010 | cytoskeleton_organization | 1 | 0 | 0 | 0 |
| GO:0007584 | response_to_nutrient | 0.5 | 0 | 0.25 | 0 |
| GO:0007623 | circadian_rhythm | 0 | 0 | 0 | -0.25 |
| GO:0009266 | response_to_temperature_stimulus | -1.5 | 2 | -2 | -1.25 |
| GO:0009408 | response_to_heat | -1 | -0.55 | 2 | -1.75 |
| GO:0009409 | response_to_cold | 0 | 2 | 2 | 2 |
| GO:0009410 | response_to_xenobiotic_stimulus | 1 | 1 | 1.25 | 0.5 |
| GO:0009411 | response_to_UV | 0 | 0.25 | 0.75 | -0.25 |
| GO:0009414 | response_to_water_deprivation | 0 | 0 | 0.25 | 0 |
| GO:0009416 | response_to_light_stimulus | 0.25 | -0.25 | 0.5 | 0 |
| GO:0009555 | pollen_development | 0 | 0 | 0 | 0 |
| GO:0009611 | response_to_wounding | -1.25 | -1.25 | -0.5 | -1.5 |
| GO:0009612 | response_to_mechanical_stimulus | -0.25 | -0.25 | 0 | -0.5 |
| GO:0009617 | response_to_bacterium | 0 | 0 | 1.5 | 0 |
| GO:0009620 | response_to_fungus | 0 | 0.25 | 1.75 | 0.5 |
| GO:0009631 | cold_acclimation | 0 | 1 | 1 | 1 |
| GO:0009636 | response_to_toxic_substance | 0 | 0 | -0.25 | 0 |
| GO:0009644 | response_to_high_light_intensity | 0.25 | 0.5 | 0 | 0.25 |
| GO:0009645 | response_to_low_light_intensity_stimulus | -0.25 | 0 | 0 | 0.25 |
| GO:0009646 | response_to_absence_of_light | 0 | 0 | 0 | 0.13 |
| GO:0009651 | response_to_salt_stress | 0 | 0.25 | 0.25 | 0.75 |
| GO:0009668 | plastid_membrane_organization | 0 | 1 | 0 | 0 |
| GO:0009687 | abscisic_acid_metabolic_process | 0.25 | 0.25 | 0 | 0.13 |
| GO:0009688 | abscisic_acid_biosynthetic_process | 0.5 | 0.25 | 0 | 0 |
| GO:0009723 | response_to_ethylene | 2 | 2 | 0.75 | 1.25 |
| GO:0009725 | response_to_hormone | 2 | 2 | 2 | 2 |
| GO:0009733 | response_to_auxin | 0.25 | 0.5 | -0.5 | 1 |
| GO:0009737 | response_to_abscisic_acid | 0.5 | 0.5 | 1.25 | 0.75 |
| GO:0009738 | abscisic_acid-activated_signaling_pathway | 0.75 | 1.25 | 1.25 | 0.5 |
| GO:0009739 | response_to_gibberellin | 1.25 | 1.25 | 0.5 | 1.5 |
| GO:0009744 | response_to_sucrose | 0 | -0.25 | -0.75 | 0 |
| GO:0009749 | response_to_glucose | 0 | -0.5 | -1.25 | -0.25 |
| GO:0009751 | response_to_salicylic_acid | 0 | 0.5 | 1.5 | 0 |
| GO:0009753 | response_to_jasmonic_acid | 0 | 0.25 | 0.5 | 0 |
| GO:0009787 | regulation_of_abscisic_acid-activated_signaling_pathway | 0.25 | 0.25 | 0.5 | 0.25 |
| GO:0009793 | embryo_development_ending_in_seed_dormancy | 0 | 0 | 0 | 0 |
| GO:0009825 | multidimensional_cell_growth | -0.25 | 0 | 0 | 0 |
| GO:0009835 | fruit_ripening | -1.5 | -1.7 | -0.25 | 0.5 |
| GO:0009836 | "fruit_ripening,_climacteric" | -0.75 | -0.5 | 0 | 0.5 |
| GO:0009845 | seed_germination | 0 | 0 | 0 | 0 |
| GO:0009856 | pollination | 0 | 0 | 0 | 0 |
| GO:0009908 | flower_development | 0 | 0 | 0 | 0 |
| GO:0009911 | positive_regulation_of_flower_development | 0 | 0 | 0 | 0 |
| GO:0010029 | regulation_of_seed_germination | 0 | 0 | 0 | 0 |
| GO:0010150 | leaf_senescence | 0 | 0 | 0 | 0 |
| GO:0010218 | response_to_far_red_light | 0 | 0 | 0 | 0 |
| GO:0010224 | response_to_UV-B | 0 | 0 | 0.25 | 0.12 |
| GO:0010294 | abscisic_acid_glucosyltransferase_activity | -1.25 | -1.5 | -1.5 | -1.25 |
| GO:0010436 | carotenoid_dioxygenase_activity | 0.75 | 0 | 0 | 0.25 |
| GO:0010467 | gene_expression | 0.5 | -0.25 | 0.5 | -0.25 |
| GO:0010628 | positive_regulation_of_gene_expression | 0.25 | -0.5 | 0.75 | 0.25 |
| GO:0010629 | negative_regulation_of_gene_expression | 0.75 | -0.25 | -0.5 | 0.5 |
| GO:0010917 | negative_regulation_of_mitochondrial_membrane_potential | 0 | 0.25 | 0.25 | 0 |
| GO:0014070 | response_to_organic_cyclic_compound | 0 | 0 | 0.5 | 0 |
| GO:0015078 | proton_transmembrane_transporter_activity | -0.75 | -1 | 0.5 | 0.25 |
| GO:0015108 | chloride_transmembrane_transporter_activity | 0 | 0 | 0.25 | 0 |
| GO:0015671 | oxygen_transport | 0.25 | -0.75 | -1 | 0 |
| GO:0016121 | carotene_catabolic_process | -0.5 | 0 | 0 | -0.13 |
| GO:0016123 | xanthophyll_biosynthetic_process | 0.25 | 0.12 | 0 | 0.25 |
| GO:0016125 | sterol_metabolic_process | 0 | 0 | 0 | 0.12 |
| GO:0016709 | "oxidoreductase_activity,_acting_on_paired_donors,_with_incorporation_or_reduction_of_molecular_oxygen,_NAD(P)H_as_one_donor,_and_incorporation_of_one_atom_of_oxygen" | 0.25 | 0.25 | 1.5 | 0.25 |
| GO:0016762 | xyloglucan:xyloglucosyl_transferase_activity | -1.5 | -1 | -0.5 | -1.75 |
| GO:0017077 | oxidative_phosphorylation_uncoupler_activity | -0.5 | -0.25 | 0.5 | -0.5 |
| GO:0017156 | calcium-ion_regulated_exocytosis | 0 | 0.25 | 0 | 0 |
| GO:0019825 | oxygen_binding | -1.5 | -1.25 | -1 | -0.5 |
| GO:0022857 | transmembrane_transporter_activity | 0.5 | 0 | 0.25 | 0 |
| GO:0023052 | signaling | 0.5 | 0.25 | 1.2 | 0.25 |
| GO:0030235 | nitric-oxide_synthase_regulator_activity | -0.75 | -0.25 | 0.5 | -0.25 |
| GO:0030247 | polysaccharide_binding | 0.25 | 0.5 | 0 | -0.5 |
| GO:0030308 | negative_regulation_of_cell_growth | 0 | 0 | 0 | 0 |
| GO:0030911 | TPR_domain_binding | 0 | 0 | 0.25 | 0 |
| GO:0031667 | response_to_nutrient_levels | 0.25 | 0 | 0.25 | 0.25 |

**Table S4**. Quality assessment appraising relevance, reliability, validity, and applicability of the evidence and risk-of-bias of the articles included in the meta-analyses.

| **Study** | **D1** | **D2** | **D3** | **D4** | **D5** | **Overall** |
| --- | --- | --- | --- | --- | --- | --- |
| Study 1 | Low | Low | Low | Low | Low | Low |
| Study 2 | Low | Low | Low | Low | Low | Some concerns |
| Study 3 | Some concerns | Low | Some concerns | Low | Low | Some concerns |
| Study 4 | Low | Low | High | Low | Some concerns | High |
| Study 5 | High | High | Low | Low | Some concerns | High |
| Study 6 | Low | High | Some concerns | Low | Low | High |
| Study 7 | Low | Some concerns | Some concerns | High | Low | High |
| Study 8 | Low | Some concerns | Some concerns | Low | Low | Some concerns |
| Study 9 | Low | Low | High | Low | Low | High |
| Study 10 | Low | Low | Low | Low | Low | Low |
| Study 11 | Low | Low | Low | Low | Low | Low |
| Study 12 | Low | Low | Low | Low | Low | Low |
| Study 13 | Low | Low | Low | Low | Low | Low |
| Study 14 | Low | Low | Low | Low | Low | Low |
| Study 15 | Some concerns | Low | Low | Some concerns | Low | Low |
| Study 16 | Low | Low | Low | Low | Low | Low |
| Study 17 | Low | Low | Low | Low | Low | Low |
| Study 18 | Low | Low | Low | Low | Low | Low |
| Study 19 | Some concerns | Some concerns | Low | Some concerns | Low | Some concerns |
| Study 20 | Low | Low | Low | Low | Low | Low |
| Study 21 | Low | Low | Low | Low | Low | Low |
| Study 22 | Low | Low | Low | Low | Low | Low |
| Study 23 | Low | Low | Low | Low | Low | Low |
| Study 24 | Low | Low | Low | Low | Low | Low |
| Study 25 | Low | Low | Low | Low | Low | Low |
| Study 26 | Low | Some concerns | Low | Some concerns | Low | Some concerns |
| Study 27 | Low | Low | Low | Low | Low | Low |
| Study 28 | Low | Low | Low | Low | Low | Low |
| Study 29 | Low | Low | Low | Low | Low | Low |
| Study 30 | Low | Low | Low | Low | Low | Low |
| Study 31 | No information | High | High | Low | High | High |
| Study 32 | Low | Low | Low | Low | Low | Low |
| Study 33 | Low | Low | Low | Low | Low | Low |
| Study 34 | Low | Low | Low | Low | Low | Low |
| Study 35 | Low | Low | Low | Low | Low | Low |
| Study 36 | Low | Low | Low | Low | Low | Low |
| Study 37 | Low | Low | Low | Low | Low | Low |
| Study 38 | Low | Some concerns | Low | Low | Some concerns | Some concerns |
| Study 39 | Low | Low | Low | Low | Low | Low |
| Study 40 | Low | Low | Low | Low | Low | Low |
| Study 41 | Low | Low | Low | Low | Low | Low |
| Study 42 | Low | Low | Low | Low | Low | Low |
| Study 43 | Low | Low | Low | Low | Low | Low |
| Study 44 | Low | Low | Low | Low | Low | Low |
| Study 45 | Low | Low | Low | Low | Low | Low |
| Study 46 | Low | Low | Low | Low | Low | Low |
| Study 47 | Low | Low | Low | Low | Low | Low |
| Study 48 | Low | Low | Low | Low | Low | Low |
| Study 49 | Low | Low | Low | Low | Low | Low |
| Study 50 | Low | Low | Low | Low | Low | Low |
| Study 51 | Low | Low | Low | Low | Low | Low |
| Study 52 | No information | High | High | Low | High | High |
| Study 53 | Low | Low | Low | Low | Low | Low |
| Study 54 | Low | Low | Low | Low | Low | Low |
| Study 55 | Low | Some concerns | Low | Low | Some concerns | Low |
| Study 56 | Low | Low | Low | Low | Low | Low |
| Study 57 | Low | Low | Low | Low | Low | Low |
| Study 58 | Low | Low | Low | Low | Low | Low |
| Study 59 | Low | Low | Low | Low | Low | Low |
| Study 60 | Low | Low | Low | Low | Low | Low |
| Study 61 | Low | Low | Low | Low | Low | Low |
| Study 62 | Some concerns | Low | Low | Low | Some concerns | Low |
| Study 63 | Low | Low | Low | Low | Low | Low |
| Study 64 | Low | Low | Low | Low | Low | Low |
| Study 65 | Low | Low | Low | Low | Low | Low |
| Study 66 | Low | Low | Low | Low | Low | Low |
| Study 67 | Low | Low | Low | Low | Low | Low |
| Study 68 | Low | Low | Low | Low | Low | Low |
| Study 69 | Low | Low | Low | Low | Low | Low |
| Study 70 | Low | Low | Low | Low | Low | Low |
| Study 71 | Low | Low | Low | Low | Low | Low |
| Study 72 | Low | Low | Low | Low | Some concerns | Low |
| Study 73 | Low | Low | Low | Low | Low | Low |
| Study 74 | Low | Low | Low | Low | Low | Low |
| Study 75 | Low | Low | Low | Low | Low | Low |
| Study 76 | Low | Low | Low | Low | Low | Low |
| Study 77 | No information | High | High | Low | High | High |
| Study 78 | Low | Low | Low | Low | Low | Low |
| Study 79 | Low | Low | Low | Low | Low | Low |
| Study 80 | No information | Some concerns | High | Some concerns | High | High |
| Study 81 | Low | Low | Low | Low | Low | Low |
| Study 82 | Low | Low | Low | Low | Low | Low |
| Study 83 | Low | Low | Low | Low | Low | Low |
| Study 84 | Low | Low | Low | Low | Low | Low |
| Study 85 | Low | Low | Low | Low | Low | Low |
| Study 86 | Low | Low | Low | Low | Low | Low |
| Study 87 | Low | Low | Low | Low | Low | Low |
| Study 88 | Low | Low | Low | Low | Low | Low |
| Study 89 | Low | Low | Low | Low | Low | Low |
| Study 90 | Low | Low | Low | Low | Low | Low |
| Study 91 | Low | Low | Low | Low | Low | Low |
| Study 92 | Low | Low | Low | Low | Low | Low |
| Study 93 | Low | Low | Low | Low | Low | Low |
| Study 94 | Low | Low | Low | Low | Low | Low |
| Study 95 | Some concerns | Low | Some concerns | Low | Some concerns | Low |
| Study 96 | Low | Low | Low | Low | Low | Low |
| Study 97 | Low | Low | Low | Low | Low | Low |
| Study 98 | Low | Low | Low | Low | Low | Low |
| Study 99 | Low | Low | Low | Low | Low | Low |
| Study 100 | Low | Low | Low | Low | Low | Low |
| Study 101 | Low | Low | Low | Low | Low | Low |
