# Supplementary figures and images for: Novel Targets for Fruit Conservation Strategies Revealed by Omics Studies: A Systematic Review and Meta‐Analysis
Source: Int J Food Sci. 2025 Oct 29;2025:9963581. doi: 10.1155/ijfo/9963581 (PMC12569611; doi:10.1155/ijfo/9963581)

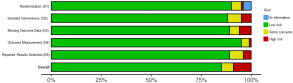

Supplement: Supplementary file 1 — Supporting Information Additional supporting information can be found online in the Supporting Information section. Table S1: Gene Ontology (GO) biological processes significantly affected by postharvest treatments and the investigated outputs in climacteric fruit in the articles included in the meta‐analyses. Table S2: Gene Ontology (GO) biological processes significantly affected by postharvest treatments and the investigated outputs in nonclimacteric fruit in the articles included in the meta‐analyses. Table S3: Odds ratio of Gene Ontology (GO) biological processes significantly affected by postharvest treatments and the investigated outputs at p < 0.05. Postharvest treatments were grouped in physical treatments, atmosphere manipulation, temperature manipulation, and edible coating. Table S4: Quality assessment appraising relevance, reliability, validity, and applicability of the evidence and risk of bias of the articles included in the meta‐analyses. Figure S1: Risk‐of‐bias assessment of the omics studies included in the systematic review. Individual and overall domains are represented by bars and risk by colors. [file IJFO-2025-9963581-s001.zip › FigureS1.pdf]
